# Nicotinic Receptors in Neurodegeneration

**DOI:** 10.2174/1570159X11311030005

**Published:** 2013-05

**Authors:** Inmaculada Posadas, Beatriz López-Hernández, Valentín Ceña

**Affiliations:** Unidad Asociada Neurodeath. CSIC-Universidad de Castilla-La Mancha, Departamento de Ciencias Médicas. Albacete, Spain and CIBERNED, Instituto de Salud Carlos III, Spain

**Keywords:** Alzheimer disease, neurodegeneration, nicotinic receptors, Parkinson disease, pharmacology, subunit composition.

## Abstract

Many studies have focused on expanding our knowledge of the structure and diversity of peripheral and central nicotinic receptors. Nicotinic acetylcholine receptors (nAChRs) are members of the Cys-loop superfamily of pentameric ligand-gated ion channels, which include GABA (A and C), serotonin, and glycine receptors. Currently, 9 alpha (α2-α10) and 3 beta (β2-β4) subunits have been identified in the central nervous system (CNS), and these subunits assemble to form a variety of functional nAChRs. The pentameric combination of several alpha and beta subunits leads to a great number of nicotinic receptors that vary in their properties, including their sensitivity to nicotine, permeability to calcium and propensity to desensitize.

In the CNS, nAChRs play crucial roles in modulating presynaptic, postsynaptic, and extrasynaptic signaling, and have been found to be involved in a complex range of CNS disorders including Alzheimer’s disease (AD), Parkinson’s disease (PD), schizophrenia, Tourette´s syndrome, anxiety, depression and epilepsy. Therefore, there is growing interest in the development of drugs that modulate nAChR functions with optimal benefits and minimal adverse effects. The present review describes the main characteristics of nAChRs in the CNS and focuses on the various compounds that have been tested and are currently in phase I and phase II trials for the treatment of neurodegenerative diseases including PD, AD and age-associated memory and mild cognitive impairment.

## 1 INTRODUCTION

The existence of nAChRs was first suggested by Langley in 1905 [1], when he showed that nicotine could stimulate denervated muscle cells. This observation led Langley to propose the concept of receptor and signal transduction [2]. Since then, there has been a breakthrough in our understanding of these receptors and they are currently well characterized. 

**Fig. (1):**
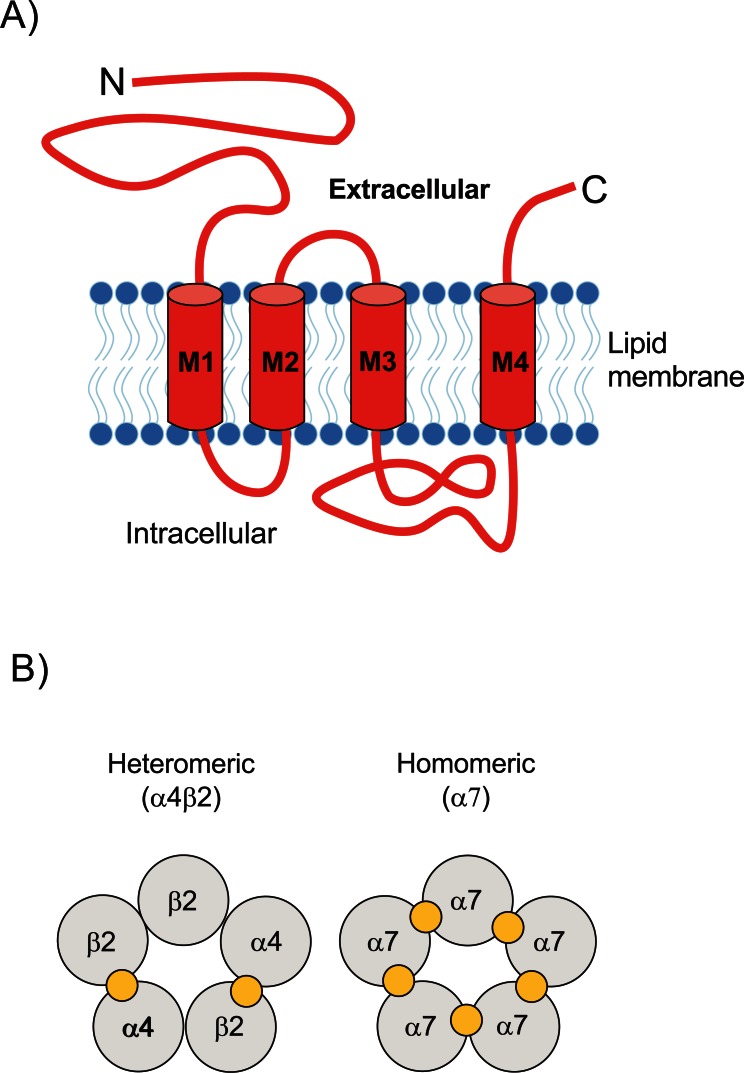
**Structure and composition of neuronal nAChR. A**) nAChR are transmembrane oligomers consisting of five subunits where each one is composed of a large amino-terminal extracellular domain, three hydrophobic transmembrane domains (M1-M3), a large intracellular loop and four hydrophobic transmembrane domains (M4). **B**) Pentameric arrangement of nAChR subunits in the neuronal α4β2 heteromeric and homomeric α7 subtypes. The localization of the ACh binding site is represented with a yellow circle.

The nAChRs are transmembrane oligomeric ligand-gated ion channels of about 300 kDa. They are composed of five subunits (Fig. **1**), and are divided into 4 subfamilies (I-IV) based on similarities of both gene structure and protein sequence [3]. To date seventeen nAChR subunits have been identified in vertebrate species (α1-α10, ß1-ß4, γ, δ and ε), and all of them except α8 are expressed in humans and in different mammalian species [4]. Assembly of the various subunits is a tightly regulated process that leads to the formation of various nAChR subtypes that differ in pharmacological properties such as agonist and antagonist sensitivity [5-7]. In mammals, nAChRs are found in both the peripheral and CNS. Peripheral nAChRs were isolated and characterized in the 1970s, while the existence of central nAChRs was unknown until the early 1980s.

In the periphery, muscular nAChRs are localized in the neuromuscular junction of somatic muscle, whereas neuronal nAChRs can be found in ganglionic cells as well as in non-neuronal cells [8,9]. Muscular nAChRs appear to have a fixed stoichiometry composed of the combination of 4 classes of subunits in which two α1 subunits co-assemble with one ß, γ and ε subunit or with one ß, δ and ε subunit in embryonic and adult muscle, respectively [4]. Conversely, neuronal nAChRs are composed of only two classes of subunits (only α and ß subunits) according to the general [2α 3ß] stoichiometry in which the two α-subunits are separated by a β-subunit [10]. In addition, homomeric receptors composed exclusively of α7 subunits have also been reported [11,12].

Activation of nAChRs by acetylcholine (ACh), nicotine or cholinergic agonist binding induces ion channel opening, allowing positively charged ions to move across it. In general, all nAChRs are permeable to Na^+^, which enters the cell, and to K^+^, which comes out the cell, but there are also some subunit combinations that are permeable to Ca^2+ ^as well [13]. This movement of cations induces the depolarization of the plasma membrane, which results in the activation of voltage-gated ion channels thus evoking an excitatory postsynaptic potential. In addition, Ca^2+^ influx mediated by some types of nAChRs can produce neurotransmitter and hormone release. The discovery that nAChRs were widely expressed in the CNS and that they were able to regulate neurotransmitter release and neuronal integration led to an increased research focus on these receptors. Interest increased even further when associations between nAChRs and neurological disorders including PD, AD, schizophrenia or epilepsy were described [13-15]. More recently, acetylcholinesterase inhibitors, used in the treatment of AD, have been reported to enhance the intrinsic action of ACh on nicotinic receptors, thus supporting a role for nAChRs in preventing neurodegeneration. Therefore, there is a growing interest in the development of drugs able to modulate nAChR functions with optimal therapeutic effects and minimal side effects.

The present review describes the main characteristics of nAChRs in the CNS and focuses on the different compounds that have been tested and are currently in phase I and phase II trials for treatment of PD, AD and age-associated memory and mild cognitive impairment.

## 2 NEURONAL ACHRS IN THE CNS

### 2.1 Subunit Composition

Neuronal nAChRs are excitatory neurotransmitter receptors that belong to the gene superfamily of ligand-gated ion channels (LGIC), which also include gamma aminobutyric acid (GABA_A_ and GABA_C_), glycine and 5-hydroxytryptamine (5HT3) receptors [8,16,17]. Neuronal nAChRs are pentameric structures formed by five subunits assembled surrounding a central aqueous pore that is permeable to Na^+^, K^+^, and Ca^2+^. All subunits consist of a large hydrophilic extracellular N-terminal domain, followed by three hydrophobic transmembrane fragments (M1-M3), a large intracellular loop, a hydrophobic transmembrane domain (M4) and the C-terminal part [17]. The general structure of nAChRs is shown in Fig. (**1a**). Twelve neuronal nAChR subunits have been cloned and their properties related to cation permeability, activation desensitization kinetics and ligand pharmacology have been characterized [18]. These subunits are designated α2-α10 and β2-β4 [8]. 

Based on binding studies, two main subfamilies of neuronal nAChRs have been identified in the CNS: low-affinity receptors, α-bungarotoxin (α-Bgtx)-sensitive, that bind agonists with low affinity (in the micromolar range) and high-affinity receptors, α-Bgtx-insensitive, that bind agonists with high affinity (in the nanomolar range). The α-Bgtx-sensitive receptors can be homopentameric (made up of the α7, α8 and α9) or heteropentameric (made up of α7α8 or α9α10). However, α-Bgtx-insensitive receptors can only be heteropentameric structures formed by a combination of α (α2-α6) and β (β2- β4) subunits [8].

The combinations of the various subunits have shown a wide range of physiological and pharmacological profiles and are differentially expressed throughout the nervous system [17]. Most neuronal nAChR subtypes are heteromeric receptors, containing at least one type of α subunit and one type of non-α subunit. Furthermore, the most abundant heteromeric receptors in the brain are composed of two α and three β subunits, in particular [(α4)_2_* (*β2)_3_], although the formation of heteromeric nAChRs containing 3 α and 2 β subunits [(α4)_3_* (*β2)_2_] has also been shown [19,20]. The [(α4)_3_* (*β2)_2_] has been described as a channel with low ionic selectivity, whereas the [(α4)_2_* (*β2)_3_] has been described as a channel with high ionic selectivity [21]. The most commonly expressed nAChR subtypes in mammalian CNS (approximately 90%) are nAChR α4β2* and α7* nAChR [4] (the structure of the most abundant nAChRs in CNS is shown in Fig. **1b**). The asterisks used in the receptor nomenclature mean that additional neuronal subunits (α5, β3) may be present in the nAChR complexes modifying the sensitivity to modulators and the pharmacological and biophysical properties of the nAChRs [4]. α5 subunits affect the pharmacological and functional properties of other nAChR subtypes, whereas β3 subunits seem to have a negative effect on the expression of the assembled β3 receptor complex [22]. In addition, it has been shown that nAChRs with the same subtype composition may display different properties depending on the subunit stoichiometries [22].

The number of agonist binding sites depends on the number of α subunits. Thus, homomeric receptors have five identical ACh-binding sites per receptor molecule (one on each subunit interface). However, in the heteromeric nAChRs, ACh and other ligands bind to two identical binding sites located at the interface between an α and a β subunits. 

### 2.2 Distribution

The regional expression of neuronal nAChRs varies among the different vertebrate species [23]. However, the available data related to the distribution of nAChRs in rodent and mammalian CNS [18] suggest that nAChRs are relatively conserved all the vertebrate species [4]. Fig. (**2**) shows the localization of nAChR subunits predominantly expressed in various rat brain regions. On the basis of the techniques currently used to examine the localization and distribution of nAChR subtypes within the CNS, it is now well established that the predominant nAChR subunits in the CNS are α4, β2, and α7, whereas α3 and β4 are the most abundant subunits in the peripheral nervous system (PNS) [24]. The most commonly expressed nAChR subtype in the CNS (90%) is the α4β2* receptor, which is characterized by its high-affinity for ACh and nicotinic agonists. In these receptors the α5 subunit is the most common additional subunit and is present in about 20% of α4β2* nAChRs expressed in striatum and cortex [22]. On the other hand the low-affinity ACh binding α7* nAChR is the other major CNS subtype [23]. 

**Fig. (2):**
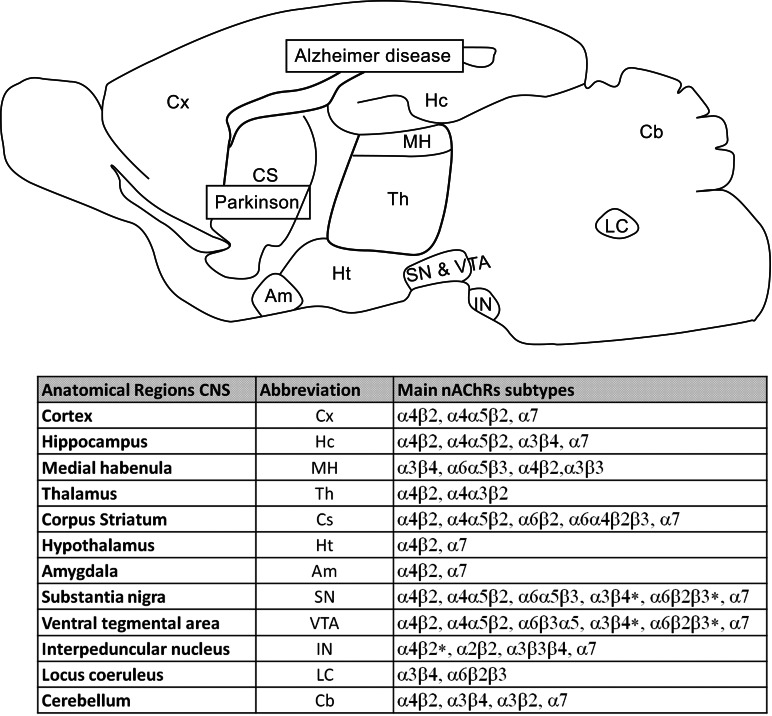
**Regional distribution of the nAChR subunits in the rodent brain.** The nAChR subunits predominantly expressed in selected CNS regions are specifically shown in the table below. The brain regions involved in Parkinson and Alzheimer diseases are indicated in the figure. Summary of the data is primarily based on references 18,22 and 23.

The β2 subunit is expressed in almost all CNS regions (Fig. **2**), where its distribution overlaps with at least one of the α (2-4, 6) subunits [25]. The α4 subunit is also widely distributed in the CNS where it co-localizes in most regions with the β2 subunit exhibiting both subunits the highest concentrations in the thalamus, hippocampus and cortex [25,26]. However, in locus coeruleus β2 but not α4 subunit is expressed [27]. Furthermore, the α4β2 subtype constitutes the main nAChR in rat brain areas such as the striatum, cortex, superior colliculus, lateral geniculate nucleus and cerebellum [28-30]. In fact, β2 or α4 subunit knockout mice lose most of their high affinity binding for nicotinic agonists in the CNS [26,31]. The α7 subunit is also widely expressed in the brain with the highest expression level in the cortex and hippocampus, whereas the subunit is absent or expressed at low levels in thalamic regions and in the basal ganglia [26,32] (Fig. **2**).

The distribution of the other nAChR subunits in the CNS appears to be restricted to localized areas of the brain, although in the neuronal population in which they are expressed, they may constitute the main subtypes of AChR. For example, α3 and β4 subunits (highly expressed in the PNS), are localized in CNS regions such as the interpeduncular nucleus, locus coeruleus and the medial and dorsal habenula [22,23,33]. The expression of the α2 nAChR appears to be expressed in small amounts in different brain regions including putamen, globus palidus, motor and somatosensory cortex and thalamus with the highest expression in the interpeduncular nucleus, where it is believed to form α2β4* and α2β2* nAChRs [23,25,26,33]. The α5 subunit is expressed in restricted CNS regions, displaying the highest expression levels in the substantia nigra, the ventral tegmental area, the medial habenula, and certain cortical and hippocampal regions [23,26]. Interestingly, the α4α5β2 subtype is specifically expressed in dopaminergic nerve terminals [23].

The distribution of subunits α6 and β3 in the CNS is also very limited. The combination of these two subunits is highly expressed in certain regions such as the substantia nigra, the ventral tegmental area (VTA), the locus coeruleus, the retina, the interpeduncular nucleus, the medial habenula and to a lesser extent in the thalamic reticular nucleus [27,34,35]. Two major combinations of α6 and β3 subunits have been identified in striatum and rat retina, α6α4β2β3 and α6β2β3 [27]. In addition, it has been reported that the α6 subunit is expressed at very high levels in dopaminergic (DA) neurons [36], which are usually localized in the striatum. In this area, the α6 subunit co-assembled with the β2 subunit, represents about 20% of the nicotinic striatal receptors while the α4β2* subtype accounts for the remaining 80% [8]. Since the α8 subunit has been only identified in the chick nervous system and the nAChRs containing α9 and/or α10 subunits have not been found in the brain [8], these subunits will not be discussed in this review. 

Knowledge of nAChR subtype distribution is important for correlating receptor subtypes and brain functions or pathologies, which would assist in creating valid animal models of human brain pathologies and finding subtype-specific therapeutic agents.

### 2.3 Function

The AChRs play an important role in many physiological and pathophysiological processes. They are widely expressed in muscle, autonomic ganglia, non-neuronal tissues and CNS. In contrast to the diversity of neuronal nAChRs previously described, muscle AChRs are composed of five subunits with fixed subunit combination: [(α1)_2_β1γδ] during development and [(α1)_2_β1εδ] in adults (Wang *et al*., 2002). In PNS nAChRs participate in the regulation of blood pressure, heart rate and gastro-intestinal stimulation among other functions. 

In the CNS, nAChRs have a dominant “presynaptic” modulatory action that regulates the release of ACh and of almost every neurotransmitter [8,18,37]. However, the presence of post-synaptic nAChRs in some areas (α7*, α4β2* and α3β4*) suggests that they may also participate in regulating cell excitability at the postsynaptic level [32]. The presence of nAChRs in cholinergic neurons indicates that they are involved in many cognitive functions such as learning and memory, motor control, reward, arousal, anxiety and central processing of pain [8,17,18,24]. In addition to the importance of nAChRs in cholinergic neurotransmission, their role as autoreceptors and heteroreceptors regulating the synaptic release of ACh and other neurotransmitters such as dopamine (DA), norepinephrine (NE), serotonin (5-hydroxytryptamine, 5-HT), glutamate (Glu), and *gamma-*-aminobutyric acid (GABA) is very relevant [25,31,38]. These neurotransmitter systems regulated by neuronal nAChRs have been proposed as potential therapeutic targets for the treatment of pain, epilepsy, and the main neurodegenerative and psychiatric disorders such as schizophrenia, anxiety, depression, AD, PD and Tourette’s syndrome [8,16,39,40]. Furthermore, it is becoming evident that the nAChRs ligands are important for the treatment of drug addiction. Systemic nicotine administration is currently the predominant smoking cessation aid [41,42].

## 3 INVOLVEMENT OF NACHRS IN NEURODEGENERATION

In the CNS, nAChRs may coexist in three distinct areas depending on their distribution on the neuron plasma membrane: receptors can be located on the presynaptic terminal, where they regulate fast synaptic transmission and modulate presynaptic transmitter release; they can be located on the axon (preterminal), where they increase the frequency of postsynaptic currents; and they can be located postsynaptically, where they mediate a general depolarization of the neuron [38,43]. According to their physiological function, two properties of nAChRs are of special interest: the inward rectification current that results in activity at hyperpolarized or resting potentials and the Na^+^ and Ca^2+^ permeability that is particularly important for presynaptic activity. In fact, Na^+^ and Ca^2+^ influx through nAChRs is sufficient to depolarize the cell, opening voltage-activated Na^+^ and Ca^2+^ channels and to activate different signaling cascades as well as Ca^2+^-dependent Cl^-^ and K^+^ currents. Moreover, as mentioned above, Na^+^ and Ca^2+^ influx through nAChRs may activate voltage-gated Ca^2+^ channels *via *the depolarization of the presynaptic membrane, leading to neurotransmitter release. This mechanism is present at cholinergic, dopaminergic, gabaergic, glutamatergic, noradrenergic, and dopaminergic synapses as well as in the autonomic ganglia including chromaffin cells [38,44]. Thus, it seems that the main role of nicotinic receptors in CNS is the regulation of neurotransmitter release, allowing ACh to participate in improving attention, learning, learned discrimination and memory functions [21,27,45].

Moreover, nicotine has also been reported to play a neuroprotective role *in vitro* and *in vivo*. In primary cultures of cortical neurons nicotine prevents the toxicity induced by various insults including glutamate [46,47], beta amyloid (Aß) peptide [48], MPP+ [49], oxidative stress [50] and rotenone [51]. Similar findings have been described in other neuronal cell types such as nigral dopaminergic neurons [52] or hippocampal neurons [53]. The mechanism involved seems to require the activation of the PI3K/Akt pathway since LY2940002, a PI3K inhibitor, reduced the protective effect of nicotine in cortical neurons. Moreover, nicotine induces upregulation of the antiapoptotic proteins Bcl-2 and Bcl-x through PI3K/Akt activation, thus preventing neuronal death [54] by inhibiting both the caspase-dependent [55] and the caspase-independent [56] apoptotic pathway. 

Nicotine neuroprotection seems to be mediated through α4β2 and α7 nAChRs, since α4β2 and α7 selective receptor antagonists have prevented the effects of nicotine [47,48,51]. Reinforcing this hypothesis, it has been shown that oxidative stress, which induces lipid peroxidation and cellular toxicity in PC12 cells, also reduces the protein level of α3 and α7 nAChR subunits [57], and Donepezil, an inhibitor of acetylcholinesterase that modulates nAChR activity, also prevented nuclear fragmentation and glutamate-induced apoptosis *via *α4β2 and α7 nAChRs [58]. Therefore, the data strongly suggest that the neuroprotection exerted by nicotine and nAChR agonists could be useful in the treatment of neurodegenerative disorders. 

The first evidence that the use of nicotinic drugs could be useful in treating neurodegenerative diseases emerged from epidemiological studies developed during early 1960s, which showed a negative correlation between smoking and the incidence of Parkinson’s disease (PD) [52,59,60]. Currently, more than 50 epidemiological studies have confirmed the low incidence of PD in smokers [61]. The observed inverse relationship between tobacco use and PD led researchers to study the role of nicotine in neurodegenerative diseases in depth. Extensive work showed that nicotine, through nAChRs activation, protected against nigrostriatal damage [62], and reduced dyskinesias, the major side-effect of L-dopa [63] which is currently the main treatment for PD. Studies performed to determine if nicotine only protected or also restored the integrity of damaged neurons, in rat and monkey PD experimental models, showed that nicotine was not able to restore the integrity of dopaminergic neurons once they have been damaged, but it attenuated ongoing neurodegenerative processes [64]. These findings suggested that nicotine, acting at nAChRs, might reduce PD progression. 

Among the many subtypes of nAChRs present in the brain, the most abundant nAChR subtype is α4β2* (more than 90%), which is detected in the cerebral cortex, thalamus, hippocampus, substantia nigra, striatum and cerebellum [22,65,66] and the highly expressed α7 homomeric subtype nAChR, found in several CNS areas including substantia nigra [29,67], seem to be particularly involved in cognitive functions [23,45]. Accumulating evidence indicates that the main nAChRs subtypes expressed in the striatum are the α4β2 and the α6β2 receptors, together with a smaller population of α7 nAChRs [68,69]. Involvement of these receptor subtypes in PD has been extensively studied. Thus, it has been shown that ACh, which is released from cholinergic interneurons in striatum, interacts with α4β2 and α6β2 to modulate dopamine release [70]. In mice and monkey models of PD and also in brains of PD patients, a greater susceptibility to neuronal damage has been observed in neurons containing the α6β2 subpopulation [63]. Moreover, a marked reduction in the number of α6β3* receptors in the dopamine pathway has been documented in PD [71]. Several studies suggest that nicotine-mediated neuroprotection against nigrostriatal damage involves various nAChR subtypes. Studies carried out in α4 nicotinic receptor knock-out mice have shown a loss of nicotine-mediated protection against 6-OHDA-induced nigro-striatal damage, thus highlighting the relevance of α4β2 receptors in this process [72]. Similarly, a role for the α6β2 subtype in neuroprotection in 6-OHDA-lesioned rats has recently been described [64]. α6* receptors, highly expressed in substantia nigra, VTA and locus coeruleus, have been shown to be necessary for the effects of dopamine on neuron activity and dopamine-dependent behavioral activities including locomotion and reinforcement [73]. α7 subtype nAChRs have also been involved in PD progression although conflicting results regarding α7 nAChR expression have been found. Administration of 6-OHDA into the striatum or the substantia nigra of mice has been shown to reduce levels of nAChRs while the levels of α7-subtype nAChRs remained unchanged [74]. Similarly, studies performed in brain tissue obtained from PD patients have shown that heterodimeric nAChRs levels decline whereas α7 nAChR levels increase selectively [63,75]. However, α7 nAChRs have been recently involved in the release of dopamine from striatum and prefrontal cortex in rats supporting a role for these nAChRs in PD [76]. It is possible that the observed up-regulation of α7 nAChRs occurs as a compensatory mechanism to maintain dopamine levels during PD progression.

Nicotine and nAChRs have been also implicated in the progressive decrease in cognitive functions and the loss of short-term memory that characterizes AD [15,22,77]. In fact, it was reported earlier that there was a massive cholinergic degeneration in AD that correlated with the decline in cognitive functions [78-80]. Moreover, nicotine administration to AD patients was shown to produce some improvements in attention and learning [81].

More recently, a significant loss of nAChRs has been observed in the cortical and hippocampal regions of brain tissue from AD patients, which may represent an early phenomenon in the progression of the disease [15,15]. Similarly, several authors have reported a decrease in the protein levels of α3, α4 and α7 nAChR subunits in the cortex and hippocampus of AD brains [82]. Moreover, a progressive loss of acetylcholinesterase activity has also been described in AD patients [83].

Among the many subtypes of nAChRs present in brain, the α7 nAChR is the most commonly expressed receptor in brain areas showing cholinergic degeneration in AD patients and its activation may promote neuronal survival [53,83,84]. In addition, Aβ peptide, the main component of the deposits found in the brains of patients with AD [83,85], interacts with nAChRs and blocks hippocampal α7 nAChRs [83,86]. Chronic perturbations of α7 nAChRs could result in neuronal dysfunction and neurodegeneration, whereas stimulation of α7 nAChRs can protect neurons from Aβ peptide-induced neuronal death [87]. Moreover, α7 nAChR activation attenuates Aß peptide synthesis, neurotoxicity and prevents loss of short-term memory in rats [87-89]. On the other hand, in human aging brain tissue, a widespread decline in nicotinic receptors has been reported, which may be related to mild cognitive impairment. This process may predispose subjects to neurodegenerative disorders such as AD and PD as mentioned above. All these data support a role for nAChRs in neurodegeneration and supports the research on pharmacological modulators of nAChRs for the treatment of neurodegenerative diseases. 

## 4 PHARMACOLOGY OF NACHRS

Nicotine, through its interaction with nAChRs, is involved in regulating cognitive and memory functions, suggesting that nAChR modulators may have the potential to alleviate cognitive impairment and prevent neurodegeneration. This potential has boosted research efforts to develop agonists for nAChRs over the last decade. Initial strategies were aimed at enhancing the concentration of ACh in brain by administering nicotine or by inhibiting ACh hydrolysis by using acetylcholinesterase inhibitors. Later, strategies were developed to modulate nAChRs in brain by using selective agonists.

The design of pharmacological strategies targeting nAChRs must take into account that these receptors may spontaneously exist in three different conformations: the resting, the active and the desensitized states. Desensitization is a characteristic process that nAChRs suffer, and it is due to prolonged exposure of nAChRs to agonists that causes a progressive, slow and reversible decline of the response following activation of the receptor. In fact, nicotine can both activate and desensitize nAChRs in a relatively short time period, leading to the question of whether desensitization plays a role in nicotine effects [90]. Thus, nicotinic ligands should be viewed as differentially stabilizing the conformational state to which they preferentially bind. Usually, agonists bind to the active state, but show higher affinity for the desensitized state, suggesting that if an agonist induces a sustained desensitization it may become an antagonist [91,92]. 

### 4.1 Nicotine and its Metabolites

Elrod, Buccafusco and Jackson were the first to report that low doses of nicotine could enhance short-term memory in monkeys [93]. Later studies have shown that nicotine improves memory in animals, healthy subjects and AD patients [79,94,95]. Thus, intravenous administration of a single dose of nicotine improved visual attention, reaction time and perception [96], and subcutaneous administration of three acute doses of nicotine to AD patients resulted in short-term improvement in learning, memory and attention performance [97]. In agreement with this, transdermal administration of 5 mg nicotine to non-smoking healthy elderly people improved short-term verbal memory functions [98]. Similarly, chronic transdermal nicotine administration in patients with AD significantly improved attention performance [97,99]. In PD animal models, nicotine improved dopaminergic markers and function in injured striatum, but failed to improve task performance in rats or monkeys [100]. The failure was believed to be due to the low doses of nicotine used and to the nonselective effects of nicotine on nAChRs. In PD patients, administration of progressively increasing doses of subcutaneous nicotine induced a marked improvement of their symptomatology [96]. Moreover, nicotine administered as a combination of transdermal patch and gum reduced rigidity, tremor and depression in patients with PD [96]. In addition, it has been recently described that acute transdermal nicotine administration improves controlled semantic processing in non-smoking PD patients, possibly *via *enhanced expectancy or an inhibitory mechanism [101].

Despite the beneficial effects observed, nicotine administration is troublesome due to its narrow therapeutic index, gastrointestinal and autonomic toxicity and a steep dose-response curve for cognitive effects, apart from its additive properties [102,103]. Other negative effects of nicotine administration include alterations in blood pressure and heart rate, mydriasis and inflammatory bowel disease. Conversely, a recent study has demonstrated that transdermal nicotine can be safely administered to non-smoking subjects with mild cognitive impairment over 6 months with improvement of primary and secondary cognitive measures of attention, memory and mental processing [104].

The observation that a single dose of nicotine with a half-life ranging from 30 minutes to 3 hours induces pro-cognitive effects that lasted over 24 hours suggests that a metabolite of nicotine may be contributing to these sustained effects. Cotinine is the main metabolite of nicotine, with a half-life of approximately 16-20 hours and a safer profile than nicotine since it does not produce cardiovascular or addictive side effects in humans [105-107]. Even at concentrations as high as 10 times those obtained from cigarette smoking, cotinine showed a safety profile with no acute or withdrawal effects and no significant effects on blood pressure [107]. Cotinine has been characterized as a weak nicotinic agonist able of evoking partial nAChR desensitization [90]. It has been reported that it improves information processing, attention and memory-related tasks function in rats and monkeys [90,108]. Moreover, cotinine induces the release of dopamine in monkey striatum [109] and it has been recently reported that chronic treatment with cotinine reduces Aß peptide aggregation and improves working memory in AD mice [106]. Taken together, these data support the cotinine molecule as a starting point for novel drug development.

Choline is a product of ACh hydrolysis by acetylcholinesterase that binds to α7 nAChRs [110] and also exhibits neuroprotective properties but with low potency [111]. JWB1-84-1 and JAY2-22-33 are two choline analogs synthesized with the expectation that they would have higher potency than choline and would be useful for cytoprotection in neurodegenerative diseases like AD. In AD transgenic mice (B6C3-Tg (APPswe, PSEN1dE9)85Dbo/J), JWB1-84-1 improved the cognitive symptoms and attention deficits associated with neurodegenerative diseases. In addition, its cytoprotective action may also be beneficial in slowing the progression of AD [112]. This compound also improved the short time memory test Delayed Matching-To-Sample (DMTS) task accuracy by aged monkeys and reversed distractor-impaired accuracies in an attention deficit model in young macaques [91]. On the other hand, JAY2-22-33, but not JWB1-84-1, significantly reduced Aß peptide levels in Neu2a cells, protected against Aß peptide toxicity in rat primary cortical neurons and delayed Aß peptide-induced paralysis in *C. elegans* [113], thus exhibiting potential for AD treatment. In addition, JAY2-22-33 exhibited similar properties to JWB1-84-1 in the attention deficit model in young macaques but with less potency [90]. Citicholine is a choline donor, involved in the biosynthesis of brain phospholipids and ACh, used in the treatment of neurodegenerative diseases. Citicholine improves memory performance in elderly subjects with minimal negative effects, and also improves the cognitive and mental performance in Alzheimer's dementia and vascular dementia [114]. The structure of nicotine, ACh, their products of metabolism and the analogs of choline are shown in Fig. (**3**).

### 4.2 Inhibitors of Acetylcholinesterase

The primary treatments for AD with proven efficacy are the acetylcholinesterase inhibitors that prevent acethylcoline degradation and prolong its activity in synapses, although their insufficient efficacy and marginal tolerability has limited their clinical utility over the years. The structures of ACh inhibitors are shown in Fig. (**4**).

Tacrine, which was shown to improve nAChRs function in AD patients during long-term treatment and seems to slow disease progression, was the first medication approved for symptomatic treatment in AD [15]. However, due to its adverse reactions and poor oral bioavaiability, tacrine has been displaced in the clinic in favor of other acetylcholinesterase inhibitors with improved safety profiles such as Donepezil, galantamine and rivastigmine.

The benefitial effects of the acethylcholinestarase inhibitors on neurodegenerative diseases have been extensively studied. A randomized, double-blind, placebo-controlled trials assessing the efficacy of treatment with acethylcholinesterase inhibitors in dementia with Lewy bodies (DLB), PD with dementia (PDD) and cognitive impairment in PD (CIPD) supports the use of acethylcholinesterase inhibitors in PDD with a positive impact on cognitive functions, behavioral disturbances and daily activities. However, the benefits on DLB and CIPD remain unclear [115]. Similarly a recent review on the clinical effectiveness and cost-effectiveness of donepezil, galantamine and rivastigmine has shown that acethylcholinesterase inhibitors display beneficial effects in alleviating AD symptoms in patients with mild-to-moderate AD, although there is a debate about the magnitude of the effect. Among the acethylcholinesterase inhibitors studied, Donepezil presents the best quality adjusted life-year (QALY) [116]. However, a recent double-blind, placebo-controlled randomized trial of acethylcholinesterase inhibitors in 5149 individuals with mild cognitive impairment shows that these drugs do not modify progression to dementia in these patients, but increase the adverse effects. The main adverse effects reported for acethylcholinesterase inhibitors include gastrointestinal effects like diarrhea, nausea and vomiting, muscle spasms, headache, syncope, dizziness, insomnia and abnormal dreams [117]. It has also been described that acetylcholinesterase inhibitors may also induce cardiovascular side-effects although their effects on the cardiovascular system are still unclear. In this regard, it has been recently described that Donepezil, galantamine and rivastigmine are not associated with cardiovascular toxicity in elderly patients with AD [118].

Donezepil, rivastigmine and galantamine have shown palliative effects on AD symptoms, a tendency to slow disease progression and improve nAChRs function during long term treatment [119], suggesting that the beneficial effects of cholinesterase inhibitors occurred, at least in part, through nAChRs activation [120]. Galantamine was approved by the FDA in 2001 for the treatment of mild to moderate dementia due to AD. It is a reversible inhibitor of acetylcholinesterase, which also acts as an allosterical ligand on the α4β2, α3β4, α6β4 and α7 nAChRs [121-123]. Increased activity of the cholinergic system in AD patients may be responsible, in part, for the improvement in cognitive functions induced by galantamine. This compound has been also shown to prevent Aß peptide-enhanced glutamate toxicity in cortical neurons, in a mechanism mediated, at least in part, by α7 nAChR [54]. In addition, activation of nAChRs by galantamine results in dopamine release in the VTA and the medial prefrontal cortex [124]. Dopamine release may be another mechanism underlying the therapeutic benefit of galantamine. 

Donepezil is one of the most common acetylcholinesterase inhibitors used for the treatment of AD. It was long thought that the beneficial effects of Donepezil were exclusively due to acetylcholinesterase inhibition, however, recent evidence suggests that Donepezil may also modulate α7 nAChRs of sustantia nigra dopaminergic neurons as well as cortical neurons and that this modulation may be relevant in neuroprotection and in improving cognition [125]. However rivastigmine, another acetylcholinesterase inhibitor used for the treatment of mild to moderate dementia due to AD or to PD has not been reported to modulate nAChRs [126-128].

### 4.3 Agonists of nAChRs

The synthesis and testing of nAChR agonists for AD and PD treatment has been the target of several pharmaceutical companies in collaboration with research groups. All of them have synthesized and tested a great number of analogs of nAChRs, and most of them have shown a good profile for neurodegenerative disorders treatment *in vitro*. In this review we have selected some of the most promising compounds from different pharmaceutical companies. The structures of some of the agonists for nAChRs that will be discussed are shown in Figs. (**5** and **6**).

ABT-418 was the first agonist for nAChRs developed by Abbott Laboratories. This compound is an analog of nicotine in which a 3-methylisoxazol-5-yl moiety replaced the pyrimidine ring of the parent compound. ABT-418 binds with high affinity to the α4β2, α2β2 and α7 subtypes, but not to the α3β4 subtype [128,129]. In animal studies, ABT-418 induced better effects on locomotor activity, learning and behavior than nicotine, with a larger therapeutic index [130]. In addition, initial studies in healthy humans suggested that ABT-418 was well tolerated, being dizziness and nausea the most frequently reported adverse effects [131]. The promising results obtained in *in vitro* experiments as well as in rats, monkeys and healthy humans, led Potter and colleagues to test ABT-418 in AD patients, showing that acute administration of this drug could improve performance on cognitive measures in AD. ABT-418 administration improved verbal learning and immediate recall as well as spatial and recognition memory [132]. Unfortunately, ABT-418 did not reproduce these encouraging results in a phase II multi-center trial, likely because of poor pharmacokinetics and tolerability issues [133]. Recently, it has been described that ABT-418 significantly improves memory in an animal model of Attention-Deficit Hyperactivity Disorder (ADHD), increases the expression of the α4 and β2 subunits in the cortex and the expression of the α4 subunit in the hippocampus providing the bases for a possible use of ABT-418 in ADHD treatment [134].

ABT-594 is a high affinity α4β2 nAChR agonist initially described as a potent, orally active and non-opiate analgesic (Holladay *et al*, 1998). Later it was shown that it increases FGF-2 expression in different rat brain regions (Belluardo *et al*., 1999) and that improved DMTS in young monkeys [135], suggesting a possible therapeutic role in neurodegenerative disorders. However, ABT-594 is a compound poorly tolerated. The most frequent adverse effects of ABT-594 include nausea, vomiting, dizziness, headache, abnormal dreams and asthenia [136,137].

ABT-089 (also known as Pozanicline) is another compound synthesized by Abbott Laboratories that acts as a partial agonist for nAChRs with very low intrinsic agonist activity. This compound binds preferentially to α4β2 and α6β2 subtype receptors and it has also been reported to exert cognition-enhancing properties and neuroprotective effects in rats and monkeys [138]. ABT-089 was effective in phase II clinical trials in AD [139] and it was also effective in a limited trial in ADHD in adults, showing improved preclinical tolerability, safety and pharmacokinetics with respect to nicotine or ABT-418 [140,141]. A recent phase II, randomized, double-blind, parallel-group, placebo-controlled pilot trial has determined that ABT-089 is generally well tolerated at doses up to 80 mg (NCT00640185). The most commonly reported adverse events of ABT-089 were nasopharyngitis, upper respiratory tract infection, and somnolence [142]. Similarly, ABT-894 is a novel subtype-selective nAChR agonist synthesized by Abbott Laboratories in collaboration with the company Neurosearch that has demonstrated a good efficacy and safety profile in a Phase II study in adults with ADHD (NCT00429091, FDA 2010). 

The anabaside analog GTS-21, also known as DMXB-A, is a partial agonist of nicotinic receptors that binds to α4β2 and α7 nAChR but only significantly activates the α7 subtype. This drug has completed Phase I trials and it has been tested for cognitive improvements in patients with AD and schizophrenia [143]. In healthy male volunteers, GTS-21 showed statistically significant enhancement of three measures of cognitive function (attention, working memory, episodic secondary memory) compared to placebo [143]. In 2006 a double blind, placebo-controlled randomized study was started to determine the safety and tolerability of GTS-21 in patients with probable AD (NTC 00414622). The study was completed in 2007 but CoMentis has not provided any information yet (see clinicaltrials.gov for more information). In phase II trial, conducted to assess whether the cognitive effects would continue during long-term administration, it was observed that the patients taking the higher dose experienced significant improvement as compared to placebo and showed increased attention/vigilance and working memory. The main adverse reactions reported were nausea and restlessness [144].

In general terms, the study of the effect of other nAChR agonists with different affinities for distinct nAChR subunits suggests that α7 ligands seem to have more pronounced effects on memory while other nAChR ligands appear to have more robust effects on attention [133]. In addition, positive allosteric modulation of α7 nAChRs has emerged as an alternative approach to the use of direct agonists that could be beneficial in certain populations where smoking nicotine is prevalent [145]. 

ABT-107 is a novel nAChR agonist that displays high affinity binding for rat and human α7 nicotinic receptors. ABT-107 exhibited nonlinear pharmacokinetics with a half-life that ranged from 7 to 10 hours achieving a steady-state concentration after 6 days of tretament. The most frequent adverse events include nausea, headache and tremor following a single dose [146]. This compound is still under development for the treatment of AD and cognitive deficits associated with schizophrenia, but preliminary results in pharmacokinetic, safety, and tolerability profiles of ABT-107 suggest that it is a good candidate for further development [146].

A582941 is another novel α7 nicotinic receptor agonist, with adequate pharmacokinetic properties and excellent distribution to the CNS. It has been reported to improve DMTS accuracies in young adult Rhesus monkeys [135], and to enhance cognitive performance, short-term recognition memory and memory consolidation in *in vivo* studies [147], thus possibly improving cognitive deficits associated with various neurodegenerative disorders. Furthermore, PNU-120596 has been recently described as a potent and selective positive allosteric modulator for the α7 subtype receptor that increases the mean open time of the nicotinic receptor ionic channel and increases the efficiency of α7 nAChRs [148,149]. PNU-120596 alone or in combination with other α7 nAChRs agonists regulates dopamine and glutamate release in rat brains [76]. Co-administration of the positive allosteric modulator (PAM) PNU-120596 with Donepezil increases its effective dose range in learning/memory-related tasks in different young and age-impaired animal models [150], suggesting that α7-nAChR-selective PAMs have potential as adjunctive treatments with acetylcholinesterase inhibitors (e.g., Donepezil) for age-related illnesses such as AD as well memory disorders not necessarily associated with advanced age. Further experiments are warranted to determine its potential for neurodegenerative diseases treatment.

TC-5619 is a highly selective agonist for α7 nAChRs recently described. TC-5619 has shown long-lasting enhancement of memory over a wide dose range in rats [151], and had positive effects across cognitive, positive, and negative symptoms of schizophrenia in animal models [152]. Phase I single rising dose clinical trial in healthy volunteers show that TC-5619 is in general well tolerated up to doses of 600 mg with no clinically noteworthy safety findings [86,153]. Other phase I and phase II clinical trials have been completed in patients with AD and ADHD but results have not been yet provided by Tagarcept Inc. (for more information visit clinicaltrials.gov and review NCT01254448, NCT01124708).

R3487/MEM3454 also named RO5313534 is an orally active α7 nAChRs agonist that also binds to human 5-HT_3_ receptor [154]. This compound increases dopamine and ACh efflux in the rat cortex and hippocampus and is effective in several behavioral paradigms in young and aged rodents [86]. In a phase IIa trial conducted in patients with mild to moderate AD, R3487/MEM3454 showed significant improvements compared with placebo being constipation the only adverse effect observed more frequently [86] (for more information visit clinicaltrials.gov NCT00884507). Another phase I trial was started in 2010 in healthy volunteers to study the effect of memantine on pharmacokinetics, safety and tolerability of RO5313534, but results obtained have not been yet provided by Hoffman-La Roche (for more information visit clinicaltrials.gov and review NCT01196065).

Another novel α-7 nAChRs partial agonist is EVP-6124, which is synthesized by EnVivo Pharmaceuticals. In oocytes, EVP-6124 increases ACh-evoked responses, and *in vivo* it significantly restores memory function in scopolamine-treated rats [155]. EVP-6124 had good brain penetration and an adequate exposure time, and significantly restored memory function and potentiated the effect of Donepezil in scopolamine-treated rats [155]. EVP-6124 is currently being tested in patients with mild to moderate AD in a phase II randomized, double-blind, placebo-controlled study (ClinicalTrials.gov identifier Nº NCT01073228) and also in a phase I double-blind, placebo- and active-controlled, 3-way crossover study to evaluate the effect of the drug on the QT interval in healthy male and female subjects (ClinicalTrials.gov identifier Nº NCT01487135). A previous randomized, double-blind, placebo-controlled, ascending-dose phase Ib safety study of three different doses of EVP-6124 in patients with mild to moderate probable AD, demonstrated that the most frequent adverse effects at the lowest dose were flatulence, nausea and headache, whereas the highest dose frequently produced gastrointestinal disorders, vomiting, upper respiratory infections and atrial fibrillation (ClinicalTrials.gov identifier NCT00766363).

As it has been previously described that Aß peptide can interact with α-7 nAChRs and suppress its activity. S24795 is a novel α-7 nAChR agonist able to interact with Aß peptide facilitating its release from the α-7 nAChR and restoring the receptor function [156]. JN403 is another novel α-7 potent and partial agonist of human nAChR [157] that inhibits the interaction of Aß peptide with α7 nAChRs and prevents the formation of Aβ/α7 nAChR complexes *in vivo* [158].

SEN12333/WAY-317538 is a novel α-7 nAChR recently described. This compound showed an excellent *in vitro* and *in vivo* profile, brain penetration and oral bioavailability, as well as *in vivo* efficacy in several behavioral cognition animal models [86,159]. These promising results makes SEN12333/WAY-317538 a possible candidate for the treatment of cognitive impairment associated with a variety of disorders including AD and schizophrenia. However, further experiments should be performed to determine its potential in humans. SIB-1508Y (Altinicline) and SIB-1553A are two nAChR agonists synthesized by SIBIA Neurosciences Inc. SIB-1508Y is selective for receptors with a β2 subunit whereas SIB-1553A binds preferentially to receptors with a β4 subunit. Although both drugs stimulate the release of dopamine at cortical and subcortical sites [160,161], SIB-1553A appears to be more potent, inducing greater release of hippocampal and prefrontal cortical ACh than either nicotine or SIB-1508Y [161]. Accordingly, the administration of SIB-1553A to MPTP-treated monkeys improved memory and attention in this PD model. The results suggest that at lower doses, SIB-1553A may be more effective in improving attention deficits whereas at higher doses, SIB-1553A may effectively improve both attention and memory performance [162]. In a non-human primate model of early PD, SIB-1553A improved performance on short cue trials in MPTP-lesioned Rhesus monkeys, and when co-administered with levodopa counteracted levodopa-induced deficits on long cue duration trials [163]. On the other hand, SIB-1508Y can also increase ACh release from the rat hippocampus without affecting striatal ACh release [164]. These findings suggest that SIB-1508Y may improve behavior by releasing multiple neurotransmitters in different brain regions. However, in a phase II trial performed on 77 individuals with early PD, this compound did not show any antiparkinsonian or cognitive-enhancing effects [165]. SIB-1765F, the racemate of SIB-1508Y, slightly improved the motor and cognitive manifestation of PD, but in combination with L-dopa strongly potentiated its effects on motor cognitive performance by increasing dopamine release from reserpine-sensitive and -insensitive pools [96].

TC-1734, also named AZD3480 or Ispronicline, was synthesized by the Winston-Salem Company in collaboration with AstraZeneca as monotherapy for mild to moderate AD [166]. TC-1734 was described as a highly selective α4β2 and α2β2 agonist able to stimulate ACh release in a dose-dependent manner in the cortex of rats, and to enhance memory in mice [167,168]. These results led to Phase I clinical trials that showed that TC-1734 had a favorable pharmacokinetic and safety profile by acute oral administration in agreement with the low toxicity previously described in mice rats and dogs [169]. Its pharmacokinetic (PK) profile (half-life of 2 h) contrasts with the long lasting improvement in working memory (18 h) suggesting that cognitive improvement extends beyond the lifetime of the compound [169]. However, later studies developed in patients with mild to moderate AD have provided mixed results. Various Phase II trials have shown significant enhancement of several cognitive measures (attention and episodic memory) compared to placebo [166,170]. However a recent Phase IIb dose-finding study did not find a statistically significant improvement in AD Assessment Scale-Cognitive Subscale (ADAS-Cog) in TC-1734-treated patients, although researchers found improvements in several secondary outcomes measures including Disability Assessment for Dementia (DAD), Mini Mental State Examination (MMSE) and AD Cooperative Study-Clinical Global Impression of Change (ADCS-CGIC), especially for the highest dose assayed [171]. Similarly, a recent study performed in 440 patients with stable schizophrenia who were active cigarette smokers has reported that TC-1734 failed to improve cognition relative to placebo [172].

In the last years, a novel approach in the regulation of nAChRs agonist has emerged consisting in modulating selectively the α4β2 nAChR *via *positive allosteric modulation. Co-administration of an allosteric agonist with a direct agonist of nAChRs has been shown to selectively enhance the potency of the direct agonist without increasing the adverse effects. This is the case of NS9283, a positive allosteric modulator of α2- and α4-containing nAChRs, that increases the analgesic efficacy of ABT-594 [173]. Moreover, NS9283 has been shown to improve performance in a rat model of episodic memory, a rat model of sustained attention, and a rat model of reference memory [174], suggesting that positive allosteric modulation of nAChRs could constitute a promising therapeutic approach to the treatment of cognitive impairment.

NS3956 is novel α4β2-selective partial nAChR agonist able to activate both HS and LS subtypes of α4β2 receptors [175]. NS3956 has been recently described to increase the release of dopamine in a concentration-dependent manner in rat striatum, and to increase rotational behavior in 6-OHDA lesioned rats [21].

## 5 FUTURE PERSPECTIVES

Despite the efforts over the last twenty years to find new therapeutic strategies based on nAChRs for neurodegenerative disorders, only varenicline, a partial agonist of the α4ß2 nAChR and a full α7 agonist nAChR, has been approved for human administration, and only as a nicotine replacement therapy [133]. Other potential indications for nAChRs modulation are still pursued with full, partial agonist ligands or allosteric modulation that may gain increased attention in the future.

To date, nAChR-targeted clinical candidates have not shown enough success to warrant further development, due to poor oral bioavailability, side effects, and/or a lack of efficacy. Therefore, a challenge in nAChRs drug design and development remains. Efforts should be aimed to improve knowledge of the expression and stoichiometry of human nAChRs subtypes that will allow us to design compounds with specific stoichiometry preferences. This would open the door to achieve specificity of action with reduced undesirable side-effects that actually include cardiovascular toxicity, emesis, seizures, and hypothermia, which result from activation of specific nAChRs in the CNS and PNS. 

In this sense, accumulating evidences indicates that α4ß2, α6ß2 and α7 nAChRs are the main subtypes involved in the improvement of attention and cognition as well as in the neuroprotective and antidepressant properties exerted by nicotine. 

## Figures and Tables

**Fig. (3):**
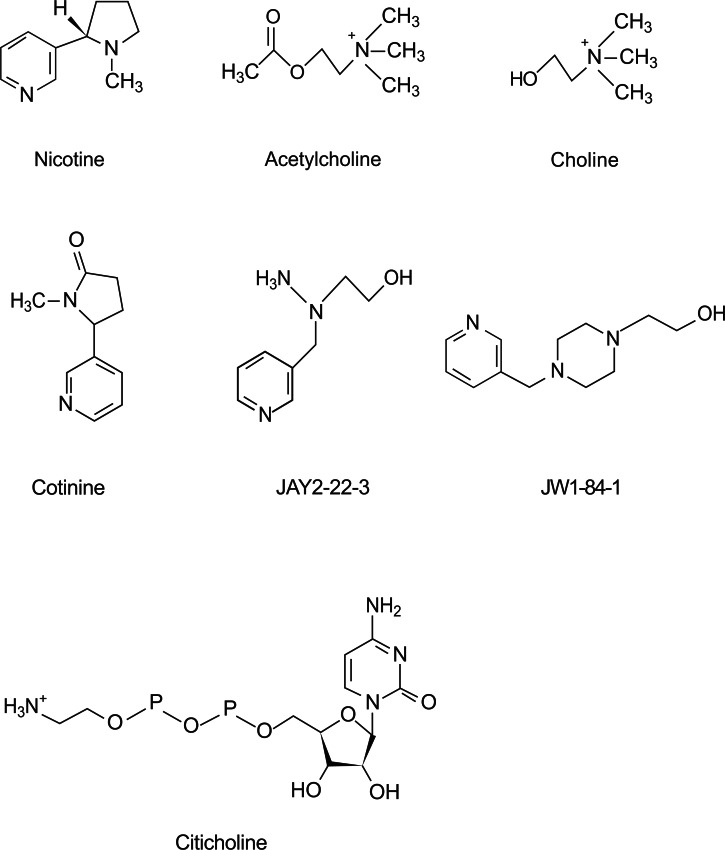
**Chemical structures of nicotine, acetylcholine and its products of metabolism.** Cotinine is one of the main products of nicotine metabolism. Choline is produced during the hydrolysis of ACh by acetylcholinesterase. JAY2-22-33 and JWB1-84-1 are two choline analogs. Citicholine is a choline donor.

**Fig. (4):**
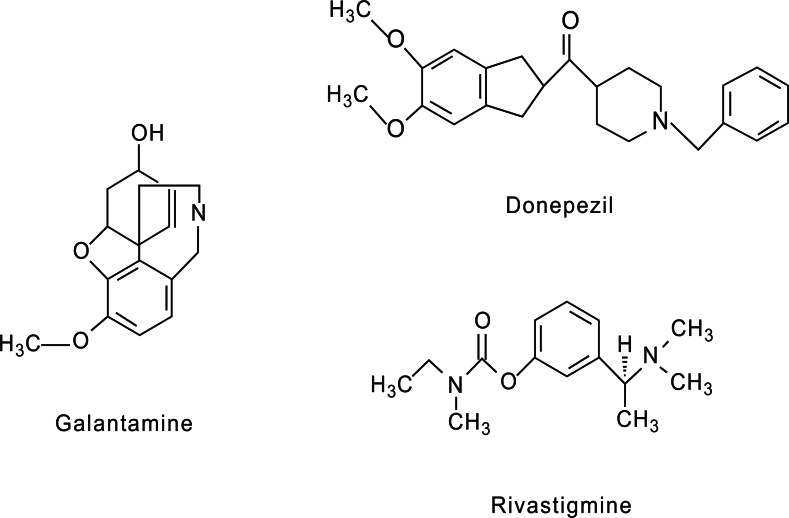
**Chemical structure of the main acetylcholinesterase inhibitors.** Galantamine and Donepezil are also described to modulate nAChRs whereas Rivastigmine seems to be active only by inhibiting acetylcholinesterase activity.

**Fig. (5):**
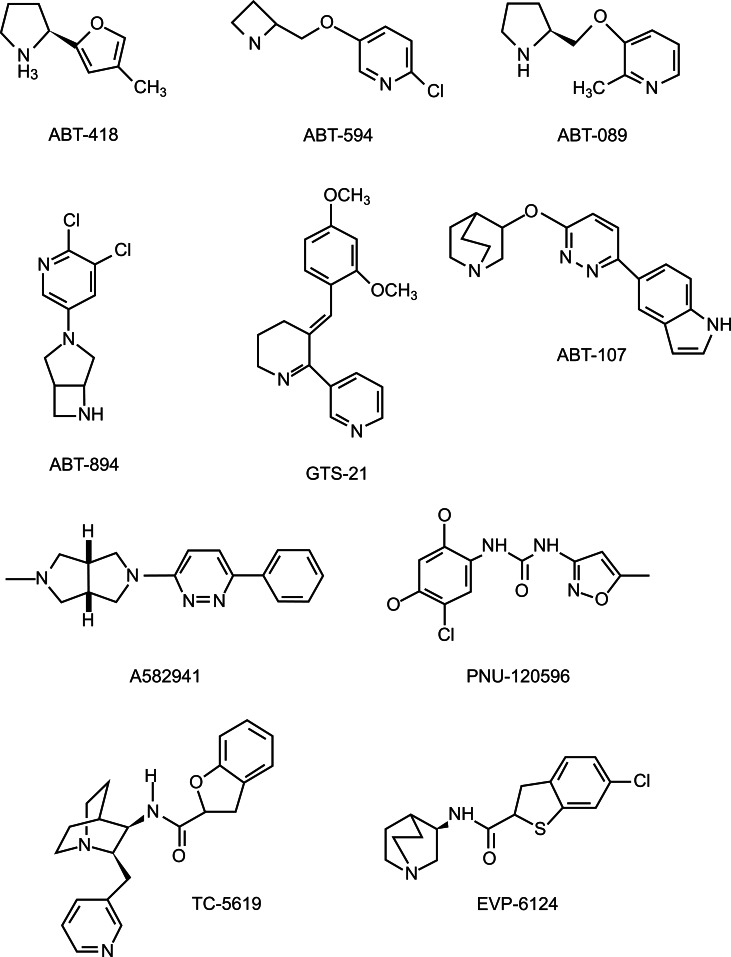
**Chemical structure of several nAChRs.** All of them produce beneficial effects on locomotor activity, learning and behavior *in vivo*.

**Fig. (6):**
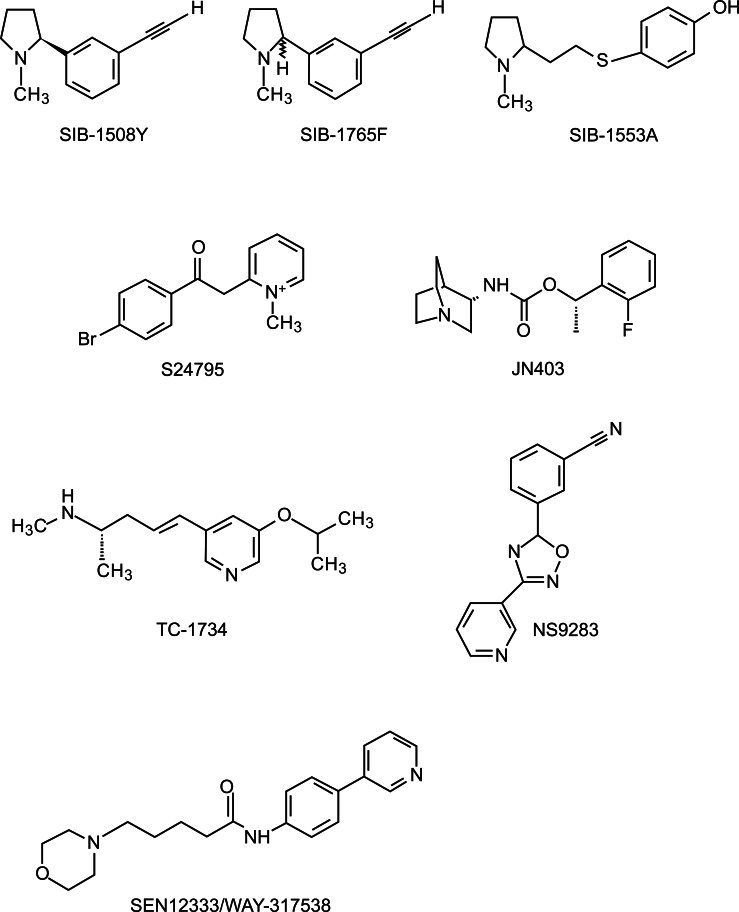
**Chemical structure of several nAChRs.** All of them produce beneficial effects on locomotor activity, learning and behavior *in vivo*.

## References

[1] Langley J N (1905). On the reaction of cells and of nerve-endings to certain poisons, chiefly as regards the reaction of striated muscle to nicotine and to curari. J. Physiol.

[2] Parascandola J, Jasensky R (1974). Origins of the receptor theory of drug action. Bull. Hist Med.

[3] Cordero-Erausquin M, Marubio L M, Klink R, Changeux J P (2000). Nicotinic receptor function: new perspectives from knockout mice. Trends Pharmacol. Sci.

[4] Millar N S, Gotti C (2009). Diversity of vertebrate nicotinic acetylcholine receptors. Neuropharmacology.

[5] Galzi J L, Changeux J P (1995). Neuronal nicotinic receptors: molecular organization and regulations. Neuropharmacology.

[6] Moroni M, Zwart R, Sher E, Cassels BK, Bermudez I (2006). alpha4beta2 nicotinic receptors with high and low acetylcholine sensitivity: pharmacology, stoichiometry, and sensitivity to 
long-term exposure to nicotine. Mol. Pharmacol.

[7] Millar N S, Harkness P C (2008). Assembly and trafficking of nicotinic acetylcholine receptors (Review). Mol. Membr. Biol.

[8] Gotti C, Clementi F (2004). Neuronal nicotinic receptors: from structure to pathology. Prog. Neurobiol.

[9] Wessler I, Kirkpatrick C J, Racke K (1999). The cholinergic 'pitfall': acetylcholine, a universal cell molecule in biological systems, including humans. Clin. Exp. Pharmacol. Physiol.

[10] Role L W (1992). Diversity in primary structure and function of neuronal nicotinic acetylcholine receptor channels. Curr. Opin. Neurobiol.

[11] Drisdel R C, Green W N (2000). Neuronal alpha-bungarotoxin receptors are alpha7 subunit homomers. J. Neurosci.

[12] Elgoyhen A B, Vetter D E, Katz E, Rothlin C V, Heinemann S F, Boulter J (2001). alpha10: a determinant of nicotinic cholinergic receptor function in mammalian vestibular and cochlear mechanosensory hair cells. Proc. Natl. Acad. Sci. U. S. A.

[13] Itier V, Bertrand D (2001). Neuronal nicotinic receptors: from protein structure to function. FEBS Lett.

[14] Newhouse P A, Kelton M (2000). Nicotinic systems in central nervous systems disease: degenerative disorders and beyond. Pharm. Acta
Helv.

[15] Nordberg A (2001). Nicotinic receptor abnormalities of Alzheimer's disease: therapeutic implications. Biol. Psychiatry.

[16] Corringer P J, Le N N, Changeux J P (2000). Nicotinic receptors at the amino acid level. Annu. Rev. Pharmacol. Toxicol.

[17] Hogg R C, Raggenbass M, Bertrand D (2003). Nicotinic acetylcholine receptors: from structure to brain function. Rev. Physiol. Biochem. Pharmacol.

[18] Jensen A A, Frolund B, Liljefors T, Krogsgaard-Larsen P (2005). Neuronal nicotinic acetylcholine receptors: structural revelations, target identifications, and therapeutic inspirations. J. Med. Chem.

[19] Zwart R, Vijverberg H P (1998). Four pharmacologically distinct subtypes of alpha4beta2 nicotinic acetylcholine receptor expressed in Xenopus laevis oocytes. Mol. Pharmacol.

[20] Nelson M E, Kuryatov A, Choi C H, Zhou Y, Lindstrom J (2003). Alternate stoichiometries of alpha4beta2 nicotinic acetylcholine receptors. Mol. Pharmacol.

[21] Rode F, Munro G, Holst D, Nielsen E O, Troelsen K B, Timmermann D B, Ronn L C, Grunnet M (2012). Positive allosteric modulation of alpha4beta2 nAChR agonist induced behaviour. Brain Res.

[22] Gotti C, Clementi F, Fornari A, Gaimarri A, Guiducci S, Manfredi I, Moretti M, Pedrazzi P, Pucci L, Zoli M (2009). Structural and functional diversity of native brain neuronal nicotinic receptors. Biochem. Pharmacol.

[23] Gotti C, Zoli M, Clementi F (2006). Brain nicotinic acetylcholine receptors: native subtypes and their relevance. Trends Pharmacol. Sci.

[24] Paterson D, Nordberg A (2000). Neuronal nicotinic receptors in the human brain. Prog. Neurobiol.

[25] Wada E, Wada K, Boulter J, Deneris E, Heinemann S, Patrick J, Swanson L W (1989). Distribution of alpha 2, alpha 3, alpha 4, and beta 2 neuronal nicotinic receptor subunit mRNAs in the central nervous system: a hybridization histochemical study in the rat. J. Comp Neurol.

[26] Picciotto M R, Caldarone B J, Brunzell D H, Zachariou V, Stevens T R, King S L (2001). Neuronal nicotinic acetylcholine receptor subunit knockout mice: physiological and behavioral phenotypes and possible clinical implications. Pharmacol. Ther.

[27] Gotti C, Moretti M, Gaimarri A, Zanardi A, Clementi F, Zoli M (2007). Heterogeneity and complexity of native brain nicotinic receptors. Biochem. Pharmacol.

[28] Picciotto M R, Zoli M (2002). Nicotinic receptors in aging and dementia. J. Neurobiol.

[29] Gotti C, Moretti M, Clementi F, Riganti L, McIntosh J M, Collins A C, Marks M J, Whiteaker P (2005). Expression of nigrostriatal alpha 6-containing nicotinic acetylcholine receptors is selectively reduced, but not eliminated, by beta 3 subunit gene deletion. Mol. Pharmacol.

[30] Turner J R, Kellar K J (2005). Nicotinic cholinergic receptors in the rat cerebellum: multiple heteromeric subtypes. J. Neurosci.

[31] Zoli M, Lena C, Picciotto M R, Changeux J P (1998). Identification of four classes of brain nicotinic receptors using beta2 mutant mice. J. Neurosci.

[32] Sher E, Chen Y, Sharples T J, Broad L M, Benedetti G, Zwart R, McPhie G I, Pearson K H, Baldwinson T, De F G (2004). Physiological roles of neuronal nicotinic receptor subtypes: new insights on the nicotinic modulation of neurotransmitter release, synaptic transmission and plasticity. Curr. Top. Med. Chem.

[33] Baddick C G, Marks M J (2011). An autoradiographic survey of mouse brain nicotinic acetylcholine receptors defined by null mutants. Biochem. Pharmacol.

[34] Kuryatov A, Olale F, Cooper J, Choi C, Lindstrom J (2000). Human alpha6 AChR subtypes: subunit composition, assembly, and pharmacological responses. Neuropharmacology.

[35] Cui C, Booker T K, Allen R S, Grady S R, Whiteaker P, Marks M J, Salminen O, Tritto T, Butt C M, Allen W R, Stitzel J A, McIntosh J M, Boulter J, Collins A C, Heinemann S F (2003). The beta3 nicotinic receptor subunit: a component of alpha-conotoxin MII-binding nicotinic acetylcholine receptors that modulate dopamine release and related behaviors. J. Neurosci.

[36] Champtiaux N, Han Z Y, Bessis A, Rossi F M, Zoli M, Marubio L, McIntosh J M, Changeux J P (2002). Distribution and pharmacology of alpha 6-containing nicotinic acetylcholine receptors analyzed with mutant mice. J. Neurosci.

[37] Dajas-Bailador F, Wonnacott S (2004). Nicotinic acetylcholine receptors and the regulation of neuronal signalling. Trends Pharmacol. Sci.

[38] Wonnacott S (1997). Presynaptic nicotinic ACh receptors. Trends Neurosci.

[39] Shytle R D, Silver A A, Lukas R J, Newman M B, Sheehan D V, Sanberg P R (2002). Nicotinic acetylcholine receptors as targets for antidepressants. Mol. Psychiatry.

[40] Ripoll N, Bronnec M, Bourin M (2004). Nicotinic receptors and schizophrenia. Curr. Med. Res. Opin.

[41] Cahill A L, Hurley J H, Fox A P (2000). Coexpression of cloned alpha(1B), beta(2a), and alpha(2)/delta subunits produces non-inactivating calcium currents similar to those found in bovine chromaffin cells. J. Neurosci.

[42] Rucktooa P, Haseler C A, van E R, Smit A B, Gallagher T, Sixma T K (2012). Structural Characterization of Binding Mode of Smoking Cessation Drugs to Nicotinic Acetylcholine Receptors through Study of Ligand Complexes with Acetylcholine-binding Protein. J. Biol. Chem.

[43] Hefft S, Hulo S, Bertrand D, Muller D (1999). Synaptic transmission at nicotinic acetylcholine receptors in rat hippocampal organotypic cultures and slices. J. Physiol.

[44] Lena C, Changeux J P (1997). Role of Ca2+ ions in nicotinic facilitation of GABA release in mouse thalamus. J. Neurosci.

[45] Levin E D (2002). Nicotinic receptor subtypes and cognitive function. J. Neurobiol.

[46] Akaike A, Tamura Y, Yokota T, Shimohama S, Kimura J (1994). Nicotine-induced protection of cultured cortical neurons against N-methyl-D-aspartate receptor-mediated glutamate cytotoxicity. Brain Res.

[47] Shimohama S, Akaike A, Kimura J (1996). Nicotine-induced
protection against glutamate cytotoxicity. Nicotinic cholinergic
receptor-mediated inhibition of nitric oxide formation. Ann. N. Y. Acad. Sci.

[48] Hunter B E, deFiebre C M, Papke R L, Kem W R, Meyer E M (1994). A novel nicotinic agonist facilitates induction of long-term potentiation in the rat hippocampus. Neurosci. Lett.

[49] Sawada H, Shimohama S, Tamura Y, Kawamura T, Akaike A, Kimura J (1996). Methylphenylpyridium ion (MPP+) enhances glutamate-induced cytotoxicity against dopaminergic neurons in cultured rat mesencephalon. J. Neurosci. Res.

[50] Cormier A, Morin C, Zini R, Tillement J P, Lagrue G (2003). Nicotine protects rat brain mitochondria against experimental injuries. Neuropharmacology.

[51] Takeuchi H, Yanagida T, Inden M, Takata K, Kitamura Y, Yamakawa K, Sawada H, Izumi Y, Yamamoto N, Kihara T, Uemura K, Inoue H, Taniguchi T, Akaike A, Takahashi R, Shimohama S (2009). Nicotinic receptor stimulation protects nigral dopaminergic neurons in rotenone-induced Parkinson's disease models. J. Neurosci. Res.

[52] Morens D M, Grandinetti A, Reed D, White L R, Ross G W (1995). Cigarette smoking and protection from Parkinson's disease: false association or etiologic clue?. Neurology.

[53] Dajas-Bailador F A, Lima P A, Wonnacott S (2000). The alpha7 nicotinic acetylcholine receptor subtype mediates nicotine protection against NMDA excitotoxicity in primary hippocampal cultures through a Ca(2+) dependent mechanism. Neuropharmacology.

[54] Kihara T, Shimohama S, Sawada H, Honda K, Nakamizo T, Shibasaki H, Kume T, Akaike A (2001). alpha 7 nicotinic receptor transduces signals to phosphatidylinositol 3-kinase to block A beta-amyloid-induced neurotoxicity. J. Biol. Chem.

[55] Liu Q, Zhao B (2004). Nicotine attenuates beta-amyloid peptide-induced neurotoxicity, free radical and calcium accumulation in hippocampal neuronal cultures. Br. J. Pharmacol.

[56] Yu W, Mechawar N, Krantic S, Quirion R (2011). alpha7 Nicotinic receptor activation reduces beta-amyloid-induced apoptosis by inhibiting caspase-independent death through phosphatidylinositol 3-kinase signaling. J. Neurochem.

[57] Guan Z Z, Zhang X, Mousavi M, Tian J Y, Unger C, Nordberg A (2001). Reduced expression of neuronal nicotinic acetylcholine receptors during the early stages of damage by oxidative stress in PC12 cells. J. Neurosci. Res.

[58] Takada Y, Yonezawa A, Kume T, Katsuki H, Kaneko S, Sugimoto H, Akaike A (2003). Nicotinic acetylcholine receptor-mediated neuroprotection by donepezil against glutamate neurotoxicity in rat cortical neurons. J. Pharmacol. Exp. Ther.

[59] Dani J A (2001). Overview of nicotinic receptors and their roles in the central nervous system. Biol. Psychiatry.

[60] Hernan M A, Zhang S M, Rueda-deCastro A M, Colditz G A, Speizer F E, Ascherio A (2001). Cigarette smoking and the incidence of Parkinson's disease in two prospective studies. Ann. Neurol.

[61] Kawamata J, Suzuki S, Shimohama S (2012). alpha7 nicotinic acetylcholine receptor mediated neuroprotection in Parkinson's disease. Curr. Drug Targets.

[62] Quik M, O'Neill M, Perez X A (2007). Nicotine neuroprotection against nigrostriatal damage: importance of the animal model. Trends Pharmacol. Sci.

[63] Bordia T, Grady S R, McIntosh J M, Quik M (2007). Nigrostriatal damage preferentially decreases a subpopulation of alpha6beta2* nAChRs in mouse, monkey, and Parkinson's disease striatum. Mol. Pharmacol.

[64] Huang L Z, Parameswaran N, Bordia T, Michael M J, Quik M (2009). Nicotine is neuroprotective when administered before but not after nigrostriatal damage in rats and monkeys. J. Neurochem.

[65] Court J, Clementi F (1995). Distribution of nicotinic subtypes in human brain. Alzheimer Dis. Assoc. Disord.

[66] Schroder H, deVos R A, Jansen E N, Birtsch C, Wevers A, Lobron C, Nowacki S, Schroder R, Maelicke A (1995). Gene expression of the nicotinic acetylcholine receptor alpha 4 subunit in the frontal cortex in Parkinson's disease patients. Neurosci. Lett.

[67] Breese C R, Marks M J, Logel J, Adams C E, Sullivan B, Collins A C, Leonard S (1997). Effect of smoking history on [3H]nicotine binding in human postmortem brain. J. Pharmacol. Exp. Ther.

[68] Grady S R, Salminen O, Laverty D C, Whiteaker P, McIntosh J M, Collins A C, Marks M J (2007). The subtypes of nicotinic acetylcholine receptors on dopaminergic terminals of mouse striatum. Biochem. Pharmacol.

[69] Millar N S, Gotti C (2009). Diversity of vertebrate nicotinic acetylcholine receptors. Neuropharmacology.

[70] Zhou F M, Wilson C J, Dani J A (2002). Cholinergic interneuron characteristics and nicotinic properties in the striatum. J. Neurobiol.

[71] Gotti C, Moretti M, Bohr I, Ziabreva I, Vailati S, Longhi R, Riganti L, Gaimarri A, McKeith I G, Perry R H, Aarsland D, Larsen J P, Sher E, Beattie R, Clementi F, Court J A (2006). Selective nicotinic acetylcholine receptor subunit deficits identified in Alzheimer's disease, Parkinson's disease and dementia with Lewy bodies by immunoprecipitation. Neurobiol. Dis.

[72] Ryan R E, Ross S A, Drago J, Loiacono R E (2001). Dose-related neuroprotective effects of chronic nicotine in 6-hydroxydopamine treated rats, and loss of neuroprotection in alpha4 nicotinic receptor subunit knockout mice. Br. J. Pharmacol.

[73] Gotti C, Guiducci S, Tedesco V, Corbioli S, Zanetti L, Moretti M, Zanardi A, Rimondini R, Mugnaini M, Clementi F, Chiamulera C, Zoli M (2010). Nicotinic acetylcholine receptors in the mesolimbic pathway: primary role of ventral tegmental area alpha6beta2* receptors in mediating systemic nicotine effects on dopamine release, locomotion, and reinforcement. J. Neurosci.

[74] Jellinger K A (2002). Recent developments in the pathology of Parkinson's disease. J. Neural Transm. Suppl.

[75] Janhunen S, Ahtee L (2007). Differential nicotinic regulation of the nigrostriatal and mesolimbic dopaminergic pathways: implications for drug development. Neurosci. Biobehav. Rev.

[76] Livingstone P D, Srinivasan J, Kew J N, Dawson L A, Gotti C, Moretti M, Shoaib M, Wonnacott S (2009). alpha7 and non-alpha7 nicotinic acetylcholine receptors modulate dopamine release *in vitro* and *in vivo* in the rat prefrontal cortex. Eur. J. Neurosci.

[77] Dournaud P, Delaere P, Hauw J J, Epelbaum J (1995). Differential correlation between neurochemical deficits, neuropathology, and cognitive status in Alzheimer's disease. Neurobiol. Aging.

[78] Bartus R T, Dean R L, Beer B, Lippa A S (1982). The cholinergic hypothesis of geriatric memory dysfunction. Science.

[79] Nordberg A, Winblad B (1986). Reduced number of [3H]nicotine and [3H]acetylcholine binding sites in the frontal cortex of Alzheimer brains. Neurosci. Lett.

[80] Perry E K, Tomlinson B E, Blessed G, Bergmann K, Gibson P H, Perry R H (1978). Correlation of cholinergic abnormalities with senile plaques and mental test scores in senile dementia. Br. Med. J.

[81] Sahakian B, Jones G, Levy R, Gray J, Warburton D (1989). The effects of nicotine on attention, information processing, and short-term memory in patients with dementia of the Alzheimer type. Br. J. Psychiatry.

[82] Guan Z Z, Zhang X, Ravid R, Nordberg A (2000). Decreased protein levels of nicotinic receptor subunits in the hippocampus and temporal cortex of patients with Alzheimer's disease. J. Neurochem.

[83] Shinotoh H, Namba H, Fukushi K, Nagatsuka S, Tanaka N, Aotsuka A, Ota T, Tanada S, Irie T (2000). Progressive loss of cortical acetylcholinesterase activity in association with cognitive decline in Alzheimer's disease: a positron emission tomography study. Ann. Neurol.

[84] Mechawar N, Saghatelyan A, Grailhe R, Scoriels L, Gheusi G, Gabellec M M, Lledo P M, Changeux J P (2004). Nicotinic receptors regulate the survival of newborn neurons in the adult olfactory bulb. Proc. Natl. Acad. Sci. U. S. A.

[85] Goedert M, Spillantini M G (2006). A century of Alzheimer's disease. Science.

[86] Toyohara J, Wu J, Hashimoto K (2010). Recent development of radioligands for imaging alpha7 nicotinic acetylcholine receptors in the brain. Curr. Top. Med. Chem.

[87] D'Andrea M R, Nagele R G (2006). Targeting the alpha 7 nicotinic acetylcholine receptor to reduce amyloid accumulation in Alzheimer's disease pyramidal neurons. Curr. Pharm. Des.

[88] Bitner R S, Nikkel A L, Markosyan S, Otte S, Puttfarcken P, Gopalakrishnan M (2009). Selective alpha7 nicotinic acetylcholine receptor activation regulates glycogen synthase kinase3beta and decreases tau phosphorylation *in vivo*. Brain Res.

[89] Buckingham S D, Jones A K, Brown L A, Sattelle D B (2009). Nicotinic acetylcholine receptor signalling: roles in Alzheimer's disease and amyloid neuroprotection. Pharmacol. Rev.

[90] Buccafusco J J, Terry A V (2009). A reversible model of the cognitive impairment associated with schizophrenia in monkeys: potential therapeutic effects of two nicotinic acetylcholine receptor agonists. Biochem. Pharmacol.

[91] Buccafusco J J, Beach J W, Terry A V (2009). Desensitization of nicotinic acetylcholine receptors as a strategy for drug development. J. Pharmacol. Exp. Ther.

[92] Romanelli M N, Gratteri P, Guandalini L, Martini E, Bonaccini C, Gualtieri F (2007). Central nicotinic receptors: structure, function, ligands, and therapeutic potential. ChemMedChem.

[93] Elrod K, Buccafusco J J, Jackson W J (1988). Nicotine enhances delayed matching-to-sample performance by primates. Life Sci.

[94] Mumenthaler M S, Yesavage J A, Taylor J L, O'Hara R, Friedman L, Lee H, Kraemer H C (2003). Psychoactive drugs and pilot performance: a comparison of nicotine, donepezil, and alcohol effects. Neuropsychopharmacology.

[95] Lendvai B, Vizi E S (2008). Nonsynaptic chemical transmission through nicotinic acetylcholine receptors. Physiol. Rev.

[96] Mihailescu S, Drucker-Colin R (2000). Nicotine, brain nicotinic receptors, and neuropsychiatric disorders. Arch. Med. Res.

[97] Jones G M, Sahakian B J, Levy R, Warburton D M, Gray J A (1992). Effects of acute subcutaneous nicotine on attention, information processing and short-term memory in Alzheimer's disease. Psychopharmacology (Berl).

[98] Min S K, Moon I W, Ko R W, Shin H S (2001). Effects of transdermal nicotine on attention and memory in healthy elderly non-smokers. Psychopharmacology (Berl).

[99] White H K, Levin E D (1999). Four-week nicotine skin patch treatment effects on cognitive performance in Alzheimer's disease. Psychopharmacology (Berl).

[100] Bordia T, Campos C, Huang L, Quik M (2008). Continuous and intermittent nicotine treatment reduces L-3,4-dihydroxyphenylalanine (L-DOPA)-induced dyskinesias in a rat model of Parkinson's disease. J. Pharmacol. Exp. Ther.

[101] Holmes A D, Copland D A, Silburn P A, Chenery H J (2011). Acute nicotine enhances strategy-based semantic processing in Parkinson's disease. Int. J. Neuropsychopharmacol.

[102] Newhouse P A, Potter A, Levin E D (1997). Nicotinic system involvement in Alzheimer's and Parkinson's diseases. Implications for therapeutics. Drugs Aging.

[103] Vaglenova J, Parameshwaran K, Suppiramaniam V, Breese CR, Pandiella N, Birru S (2008). Long-lasting teratogenic effects of nicotine on cognition: gender specificity and role of AMPA receptor function. Neurobiol. Learn. Mem.

[104] Newhouse P, Kellar K, Aisen P, White H, Wesnes K, Coderre E, Pfaff A, Wilkins H, Howard D, Levin E D (2012). Nicotine treatment of mild cognitive impairment: a 6-month double-blind pilot clinical trial. Neurology.

[105] Benowitz N L, Jacob P (1994). Metabolism of nicotine to cotinine studied by a dual stable isotope method. Clin. Pharmacol. Ther.

[106] Echeverria V, Zeitlin R, Burgess S, Patel S, Barman A, Thakur G, Mamcarz M, Wang L, Sattelle D B, Kirschner D A, Mori T, Leblanc R M, Prabhakar R, Arendash G W (2011). Cotinine reduces amyloid-beta aggregation and improves memory in Alzheimer's disease mice. J. Alzheimers. Dis.

[107] Hatsukami D K, Grillo M, Pentel P R, Oncken C, Bliss R (1997). Safety of cotinine in humans: physiologic, subjective, and cognitive effects. Pharmacol. Biochem. Behav.

[108] Terry A V, Hernandez C M, Hohnadel E J, Bouchard K P, Buccafusco J J (2005). Cotinine, a neuroactive metabolite of nicotine: potential for treating disorders of impaired cognition. CNS Drug Rev.

[109] O'Leary K, Parameswaran N, McIntosh J M, Quik M (2008). Cotinine selectively activates a subpopulation of alpha3/alpha6beta2 nicotinic receptors in monkey striatum. J. Pharmacol. Exp. Ther.

[110] Alkondon M, Pereira E F, Cortes W S, Maelicke A, Albuquerque E X (1997). Choline is a selective agonist of alpha7 nicotinic acetylcholine receptors in the rat brain neurons. Eur. J. Neurosci.

[111] Jonnala R R, Graham J H, Terry A V, Beach J W, Young J A, Buccafusco J J (2003). Relative levels of cytoprotection produced by analogs of choline and the role of alpha7-nicotinic acetylcholine receptors. Synapse.

[112] Sood A, Warren B J, Webster S J, Terry A V, Buccafusco J J (2007). The effects of JWB1-84-1 on memory-related task performance by amyloid Abeta transgenic mice and by young and aged monkeys. Neuropharmacology.

[113] Keowkase R, Aboukhatwa M, Adam B L, Beach J W, Terry A V, Buccafussco J J, Luo Y (2010). Neuroprotective effects and mechanism of cognitive-enhancing choline analogs JWB 1-84-1 and JAY 2-22-33 in neuronal culture and *Caenorhabditis elegans*. Mol. Neurodegener.

[114] Putignano S, Gareri P, Castagna A, Cerqua G, Cervera P, Cotroneo A M, Fiorillo F, Grella R, Lacava R, Maddonni A, Marino S, Pluderi A, Putignano D, Rocca F (2012). Retrospective and observational study to assess the efficacy of citicoline in elderly patients suffering from stupor related to complex geriatric syndrome. Clin. Interv. Aging.

[115] Rolinski M, Fox C, Maidment I, McShane R (2012). Cholinesterase inhibitors for dementia with Lewy bodies, Parkinson's disease dementia and cognitive impairment in Parkinson's disease. Cochrane. Database. Syst. Rev.

[116] Bond M, Rogers G, Peters J, Anderson R, Hoyle M, Miners A, Moxham T, Davis S, Thokala P, Wailoo A, Jeffreys M, Hyde C (2012). The effectiveness and cost-effectiveness of donepezil,
galantamine, rivastigmine and memantine for the treatment of
Alzheimer's disease (review of Technology Appraisal No. 111): a
systematic review and economic model. Health Technol. Assess.

[117] Russ T C, Morling J R (2012). Cholinesterase inhibitors for mild cognitive impairment. Cochrane. Database. Syst. Rev.

[118] Isik A T, Yildiz G B, Bozoglu E, Yay A, Aydemir E (2012). Cardiac safety of donepezil in elderly patients with Alzheimer disease. Intern. Med.

[119] Giacobini E (2000). Cholinesterase inhibitor therapy stabilizes symptoms of Alzheimer disease. Alzheimer Dis. Assoc. Disord.

[120] Maelicke A, Schrattenholz A, Samochocki M, Radina M, Albuquerque E X (2000). Allosterically potentiating ligands of nicotinic receptors as a treatment strategy for Alzheimer's disease. Behav.
Brain Res.

[121] Samochocki M, Zerlin M, Jostock R, Groot Kormelink P J, Luyten W H, Albuquerque E X, Maelicke A (2000). Galantamine is an allosterically potentiating ligand of the human alpha4/beta2 nAChR. Acta Neurol. Scand. Suppl.

[122] Kihara T, Sawada H, Nakamizo T, Kanki R, Yamashita H, Maelicke A, Shimohama S (2004). Galantamine modulates nicotinic receptor and blocks Abeta-enhanced glutamate toxicity. Biochem. Biophys. Res. Commun.

[123] Lopes C, Pereira E F, Wu H Q, Purushottamachar P, Njar V, Schwarcz R, Albuquerque E X (2007). Competitive antagonism between the nicotinic allosteric potentiating ligand galantamine and kynurenic acid at alpha7* nicotinic receptors. J. Pharmacol. Exp. Ther.

[124] Schilstrom B, Ivanov V B, Wiker C, Svensson T H (2007). Galantamine enhances dopaminergic neurotransmission *in vivo via* allosteric potentiation of nicotinic acetylcholine receptors. Neuropsychopharmacology.

[125] Shen H, Kihara T, Hongo H, Wu X, Kem W R, Shimohama S, Akaike A, Niidome T, Sugimoto H (2010). Neuroprotection by donepezil against glutamate excitotoxicity involves stimulation of alpha7 nicotinic receptors and internalization of NMDA receptors. Br. J. Pharmacol.

[126] Emre M, Aarsland D, Albanese A, Byrne E J, Deuschl G, De Deyn P P, Durif F, Kulisevsky J, van L T, Lees A, Poewe W, Robillard A, Rosa M M, Wolters E, Quarg P, Tekin S, Lane R (2004). Rivastigmine for dementia associated with Parkinson's disease. N. Engl. J. Med.

[127] Finkel S I (2004). Effects of rivastigmine on behavioral and psychological symptoms of dementia in Alzheimer's disease. Clin. Ther.

[128] Papke R L, Thinschmidt J S, Moulton B A, Meyer E M, Poirier A (1997). Activation and inhibition of rat neuronal nicotinic receptors by ABT-418. Br. J. Pharmacol.

[129] Arneric S P, Sullivan J P, Briggs C A, Donnelly-Roberts D, Anderson D J, Raszkiewicz J L, Hughes M L, Cadman E D, Adams P, Garvey D S (1994). (S)-3-methyl-5-(1-methyl-2-pyrrolidinyl) isoxazole (ABT 418): a novel cholinergic ligand with cognition-enhancing and anxiolytic activities: I. *In vitro* characterization. J. Pharmacol. Exp. Ther.

[130] Decker M W, Curzon P, Brioni J D, Arneric S P (1994). Effects of ABT-418, a novel cholinergic channel ligand, on place learning in septal-lesioned rats. Eur. J. Pharmacol.

[131] Wilens T E, Biederman J, Spencer T J, Bostic J, Prince J, Monuteaux M C, Soriano J, Fine C, Abrams A, Rater M, Polisner D (1999). A pilot controlled clinical trial of ABT-418, a cholinergic agonist, in the treatment of adults with attention deficit hyperactivity disorder. Am. J. Psychiatry.

[132] Potter A, Corwin J, Lang J, Piasecki M, Lenox R, Newhouse P A (1999). Acute effects of the selective cholinergic channel activator (nicotinic agonist) ABT-418 in Alzheimer's disease. Psychopharmacology (Berl).

[133] Terry A V, Decker M W (2011). Neurobiology of nAChRs and
cognition: a mini review of Dr. Jerry J. Buccafusco's contributions
over a 25 year career. Biochem. Pharmacol.

[134] Guo T, Yang C, Guo L, Liu K (2012). A comparative study of the effects of ABT-418 and methylphenidate on spatial memory in an animal model of ADHD. Neurosci. Lett.

[135] Buccafusco J J, Terry A V, Decker M W, Gopalakrishnan M (2007). Profile of nicotinic acetylcholine receptor agonists ABT-594 and A-582941, with differential subtype selectivity, on delayed matching accuracy by young monkeys. Biochem. Pharmacol.

[136] Dutta S, Hosmane B S, Awni W M (2012). Population analyses of efficacy and safety of ABT-594 in subjects with diabetic peripheral neuropathic pain. AAPS J.

[137] Rowbotham M C, Duan W R, Thomas J, Nothaft W, Backonja M M (2009). A randomized, double-blind, placebo-controlled trial evaluating the efficacy and safety of ABT-594 in patients with diabetic peripheral neuropathic pain. Pain.

[138] Decker M W, Bannon A W, Curzon P, Gunther K L, Brioni J D, Holladay M W, Lin N H, Li Y, Daanen J F, Buccafusco J J, Prendergast M A, Jackson W J, Arneric S P (1997). ABT-089 [2-methyl-3-(2-(S)-pyrrolidinylmethoxy)pyridine dihydrochloride]: II. A novel cholinergic channel modulator with effects on cognitive performance in rats and monkeys. J. Pharmacol. Exp. Ther.

[139] Arneric S P, Holladay M, Williams M (2007). Neuronal nicotinic receptors: a perspective on two decades of drug discovery research. Biochem. Pharmacol.

[140] Wilens T E, Verlinden M H, Adler L A, Wozniak P J, West S A (2006). ABT-089, a neuronal nicotinic receptor partial agonist, for the treatment of attention-deficit/hyperactivity disorder in adults: results of a pilot study. Biol. Psychiatry.

[141] Wilens T E, Decker M W (2007). Neuronal nicotinic receptor agonists for the treatment of attention-deficit/hyperactivity disorder: focus on cognition. Biochem. Pharmacol.

[142] Bain E E, Apostol G, Sangal R B, Robieson W Z, McNeill D L, Abi-Saab W M, Saltarelli M D (2012). A randomized pilot study of the efficacy and safety of ABT-089, a novel alpha4beta2 neuronal nicotinic receptor agonist, in adults with attention-deficit/hyperactivity disorder. J. Clin. Psychiatry.

[143] Kitagawa H, Takenouchi T, Azuma R, Wesnes K A, Kramer W G, Clody D E, Burnett A L (2003). Safety, pharmacokinetics, and effects on cognitive function of multiple doses of GTS-21 in healthy, male volunteers. Neuropsychopharmacology.

[144] Freedman R, Olincy A, Buchanan R W, Harris J G, Gold J M, Johnson L, Allensworth D, Guzman-Bonilla A, Clement B, Ball M P, Kutnick J, Pender V, Martin L F, Stevens K E, Wagner B D, Zerbe G O, Soti F, Kem W R (2008). Initial phase 2 trial of a nicotinic agonist in schizophrenia. Am. J. Psychiatry.

[145] Wallace T L, Porter R H (2011). Targeting the nicotinic alpha7 acetylcholine receptor to enhance cognition in disease. Biochem. Pharmacol.

[146] Othman A A, Lenz R A, Zhang J, Li J, Awni W M, Dutta S (2011). Single- and multiple-dose pharmacokinetics, safety, and tolerability of the selective alpha7 neuronal nicotinic receptor agonist, ABT-107, in healthy human volunteers. J. Clin. Pharmacol.

[147] Tietje K R, Anderson D J, Bitner R S, Blomme E A, Brackemeyer P J, Briggs C A, Browman K E, Bury D, Curzon P, Drescher K U, Frost J M, Fryer R M, Fox G B, Gronlien J H, Hakerud M, Gubbins E J, Halm S, Harris R, Helfrich R J, Kohlhaas K L, Law D, Malysz J, Marsh K C, Martin R L, Meyer M D, Molesky A L, Nikkel A L, Otte S, Pan L, Puttfarcken P S, Radek R J, Robb H M, Spies E, Thorin-Hagene K, Waring J F, Ween H, Xu H, Gopalakrishnan M, Bunnelle W H (2008). Preclinical characterization of A-582941: a novel alpha7 neuronal nicotinic receptor agonist with broad spectrum cognition-enhancing properties. CNS. Neurosci. Ther.

[148] Hurst R S, Hajos M, Raggenbass M, Wall T M, Higdon N R, Lawson J A, Rutherford-Root K L, Berkenpas M B, Hoffmann W E, Piotrowski D W, Groppi V E, Allaman G, Ogier R, Bertrand S, Bertrand D, Arneric S P (2005). A novel positive allosteric modulator of the alpha7 neuronal nicotinic acetylcholine receptor: *in vitro* and *in vivo* characterization. J. Neurosci.

[149] Sharma G, Grybko M, Vijayaraghavan S (2008). Action potential-independent and nicotinic receptor-mediated concerted release of multiple quanta at hippocampal CA3-mossy fiber synapses. J. Neurosci.

[150] Callahan P M, Hutchings E J, Kille N J, Chapman J M, Terry A V (2013). Positive allosteric modulator of alpha 7 nicotinic-acetylcholine receptors, PNU-120596 augments the effects of donepezil on learning and memory in aged rodents and non-human primates. Neuropharmacology.

[151] Hauser T A, Kucinski A, Jordan K G, Gatto G J, Wersinger S R, Hesse R A, Stachowiak E K, Stachowiak M K, Papke R L, Lippiello P M, Bencherif M (2009). TC-5619: an alpha7 neuronal nicotinic receptor-selective agonist that demonstrates efficacy in animal models of the positive and negative symptoms and cognitive dysfunction of schizophrenia. Biochem. Pharmacol.

[152] Mazurov A A, Kombo D C, Hauser T A, Miao L, Dull G, Genus J F, Fedorov N B, Benson L, Sidach S, Xiao Y, Hammond P S, James J W, Miller C H, Yohannes D (2012). Discovery of (2S,3R)-N-[2-(Pyridin-3-ylmethyl)-1-azabicyclo[2.2.2]
oct-3-yl]benzo[b]furan-2-car boxamide (TC-5619), a selective
alpha7 nicotinic acetylcholine receptor agonist, for the treatment of
cognitive disorders. J. Med. Chem.

[153] Lieberman J A, Dunbar G, Segreti A C, Girgis R R, Seoane F, Beaver J S, Duan N, Hosford D A (2013). A Randomized Exploratory Trial of an Alpha-7 Nicotinic Receptor Agonist (TC-5619) for Cognitive Enhancement in Schizophrenia. Neuropsychopharmacology.

[154] Rezvani A H, Eddins D, Slade S, Hampton D S, Christopher N C, Petro A, Horton K, Johnson M, Levin E D (2008). Neonatal 6-hydroxydopamine lesions of the frontal cortex in rats: persisting effects on locomotor activity, learning and nicotine self-administration. Neuroscience.

[155] Prickaerts J, van Goethem N P, Chesworth R, Shapiro G, Boess F G, Methfessel C, Reneerkens O A, Flood D G, Hilt D, Gawryl M, Bertrand S, Bertrand D, Konig G (2012). EVP-6124, a novel and selective alpha7 nicotinic acetylcholine receptor partial agonist, improves memory performance by potentiating the acetylcholine response of alpha7 nicotinic acetylcholine receptors. Neuropharmacology.

[156] Wang H Y, Stucky A, Liu J, Shen C, Trocme-Thibierge C, Morain P (2009). Dissociating beta-amyloid from alpha 7 nicotinic acetylcholine receptor by a novel therapeutic agent, S 24795, normalizes alpha 7 nicotinic acetylcholine and NMDA receptor function in Alzheimer's disease brain. J. Neurosci.

[157] Arias H R, Gu R X, Feuerbach D, Wei D Q (2010). Different interaction between the agonist JN403 and the competitive antagonist methyllycaconitine with the human alpha7 nicotinic acetylcholine receptor. Biochemistry.

[158] Lilja A M, Porras O, Storelli E, Nordberg A, Marutle A (2011). Functional interactions of fibrillar and oligomeric amyloid-beta with alpha7 nicotinic receptors in Alzheimer's disease. J.
Alzheimers Dis.

[159] Haydar S N, Ghiron C, Bettinetti L, Bothmann H, Comery T A, Dunlop J, La R S, Micco I, Pollastrini M, Quinn J, Roncarati R, Scali C, Valacchi M, Varrone M, Zanaletti R (2009). SAR and biological evaluation of SEN12333/WAY-317538: Novel alpha 7 nicotinic acetylcholine receptor agonist. Bioorg. Med. Chem.

[160] Cosford N D, Bleicher L, Herbaut A, McCallum J S, Vernier J M, Dawson H, Whitten J P, Adams P, Chavez-Noriega L, Correa L D, Crona J H, Mahaffy L S, Menzaghi F, Rao T S, Reid R, Sacaan A I, Santori E, Stauderman K A, Whelan K, Lloyd G K, McDonald I A (1996). (S)-(-)-5-ethynyl-3-(1-methyl-2-pyrrolidinyl)pyridine maleate (SIB-1508Y): a novel anti-parkinsonian agent with selectivity for neuronal nicotinic acetylcholine receptors. J. Med. Chem.

[161] Vernier J M, El-Abdellaoui H, Holsenback H, Cosford N D, Bleicher L, Barker G, Bontempi B, Chavez-Noriega L, Menzaghi F, Rao T S, Reid R, Sacaan A I, Suto C, Washburn M, Lloyd G K, McDonald I A (1999). 4-[[2-(1-Methyl-2-pyrrolidinyl)ethyl]thio]phenol hydrochloride (SIB-1553A): a novel cognitive enhancer with selectivity for neuronal nicotinic acetylcholine receptors. J. Med. Chem.

[162] Schneider J S, Tinker J P, Menzaghi F, Lloyd G K (2003). The subtype-selective nicotinic acetylcholine receptor agonist SIB-1553A improves both attention and memory components of a spatial working memory task in chronic low dose 1-methyl-4-phenyl-1,2,3,6-tetrahydropyridine-treated monkeys. J. Pharmacol. Exp. Ther.

[163] Decamp E, Schneider J S (2009). Interaction between nicotinic and dopaminergic therapies on cognition in a chronic Parkinson model. Brain Res.

[164] Rao T S, Adams P B, Correa L D, Santori E M, Sacaan A I, Reid R T, Cosford N D (2008). Pharmacological characterization of (S)-(2)-5-ethynyl-3-(1-methyl-2-pyrrolidinyl)pyridine HCl (SIB-1508Y, Altinicline), a novel nicotinic acetylcholine receptor agonist. Brain Res.

[165] The Parkinson Study Group (2006). Randomized placebo-controlled study
of the nicotinic agonist SIB-1508Y in Parkinson disease. Neurology.

[166] Dunbar G, Boeijinga P H, Demazieres A, Cisterni C, Kuchibhatla R, Wesnes K, Luthringer R (2007). Effects of TC-1734 (AZD3480), a selective neuronal nicotinic receptor agonist, on cognitive performance and the EEG of young healthy male volunteers. Psychopharmacology (Berl).

[167] Obinu M C, Reibaud M, Miquet J M, Pasquet M, Rooney T (2002). Brain-selective stimulation of nicotinic receptors by TC-1734 enhances ACh transmission from frontoparietal cortex and memory in rodents. Prog. Neuropsychopharmacol. Biol. Psychiatry.

[168] Frolich L, Ashwood T, Nilsson J, Eckerwall G (2011). Effects of AZD3480 on cognition in patients with mild-to-moderate Alzheimer's disease: a phase IIb dose-finding study. J. Alzheimers
Dis.

[169] Gatto G J, Bohme G A, Caldwell W S, Letchworth S R, Traina V M, Obinu M C, Laville M, Reibaud M, Pradier L, Dunbar G, Bencherif M (2004). TC-1734: an orally active neuronal nicotinic acetylcholine receptor modulator with antidepressant, neuroprotective and long-lasting cognitive effects. CNS. Drug Rev.

[170] Dunbar G C, Kuchibhatla R V, Lee G (2011). A randomized double-blind study comparing 25 and 50 mg TC-1734 (AZD3480) with placebo, in older subjects with age-associated memory impairment. J. Psychopharmacol.

[171] Frolich L, Ashwood T, Nilsson J, Eckerwall G (2011). Effects of AZD3480 on cognition in patients with mild-to-moderate Alzheimer's disease: a phase IIb dose-finding study. J. Alzheimers
Dis.

[172] Velligan D, Brenner R, Sicuro F, Walling D, Riesenberg R, Sfera A, Merideth C, Sweitzer D, Jaeger J (2012). Assessment of the effects of AZD3480 on cognitive function in patients with schizophrenia. Schizophr. Res.

[173] Lee C H, Zhu C, Malysz J, Campbell T, Shaughnessy T, Honore P, Polakowski J, Gopalakrishnan M (2011). alpha4beta2 neuronal nicotinic receptor positive allosteric modulation: an approach for improving the therapeutic index of alpha4beta2 nAChR agonists in pain. Biochem. Pharmacol.

[174] Timmermann D B, Sandager-Nielsen K, Dyhring T, Smith M, Jacobsen A M, Nielsen E O, Grunnet M, Christensen J K, Peters D, Kohlhaas K, Olsen G M, Ahring P K (2012). Augmentation of cognitive function by NS9283, a stoichiometry-dependent positive allosteric modulator of alpha2- and alpha4-containing nicotinic acetylcholine receptors. Br. J. Pharmacol.

[175] Andreasen J T, Nielsen E O, Christensen J K, Olsen G M, Peters D, Mirza N R, Redrobe J P (2011). Subtype-selective nicotinic acetylcholine receptor agonists enhance the responsiveness to citalopram and reboxetine in the mouse forced swim test. J. Psychopharmacol.

